# Covalent and Non-covalent Functionalized Nanomaterials for Environmental Restoration

**DOI:** 10.1007/s41061-022-00397-3

**Published:** 2022-08-11

**Authors:** Shizhong Zhang, Sumeet Malik, Nisar Ali, Adnan Khan, Muhammad Bilal, Kashif Rasool

**Affiliations:** 1 Key Laboratory for Palygorskite Science and Applied Technology of Jiangsu Province, National and Local Joint Engineering Research Center for Mineral Salt Deep Utilization, Huaiyin Institute of Technology, Huai’an, 223003 China; 2 Institute of Chemical Sciences, University of Peshawar, Khyber Pakhtunkhwa, 25120 Pakistan; 3 School of Life Science and Food Engineering, Huaiyin Institute of Technology, Huai’an, 223003 China; 4 Qatar Environment and Energy Research Institute, Hamad Bin Khalifa University (HBKU), Qatar Foundation, P.O. Box 5824, Doha, Qatar

**Keywords:** Contaminants, Photocatalysis, Functionalized nanomaterials, Quantum dots, Carbon nanomaterials, Graphene nanomaterials

## Abstract

Nanotechnology has emerged as an extraordinary and rapidly developing discipline of science. It has remolded the fate of the whole world by providing diverse horizons in different fields. Nanomaterials are appealing because of their incredibly small size and large surface area. Apart from the naturally occurring nanomaterials, synthetic nanomaterials are being prepared on large scales with different sizes and properties. Such nanomaterials are being utilized as an innovative and green approach in multiple fields. To expand the applications and enhance the properties of the nanomaterials, their functionalization and engineering are being performed on a massive scale. The functionalization helps to add to the existing useful properties of the nanomaterials, hence broadening the scope of their utilization. A large class of covalent and non-covalent functionalized nanomaterials (FNMs) including carbons, metal oxides, quantum dots, and composites of these materials with other organic or inorganic materials are being synthesized and used for environmental remediation applications including wastewater treatment. This review summarizes recent advances in the synthesis, reporting techniques, and applications of FNMs in adsorptive and photocatalytic removal of pollutants from wastewater. Future prospects are also examined, along with suggestions for attaining massive benefits in the areas of FNMs.

## Introduction

Water, which covers 70% of the earth’s surface, is a basic necessity for the life of every individual on earth. Nevertheless, extreme ecological contamination jeopardizes human well-being [1, 2]. Environmental contamination has become a major issue in industrialized and developing nations as a consequence of industrialization [3]. Remediation of water, air, and soil contamination is of great concern, especially for developing nations [4]. The fundamental contaminants in water (including surface water, groundwater, and tap water) include heavy metal ions, inorganic compounds (nitrates, chlorides, phosphates, etc.), dyes (synthetic as well as natural), surfactants, pharmaceuticals, pesticides, and numerous other complex compounds [5, 6]. The major sources of heavy metals include industrial effluents, agricultural operations, mining, and metallurgical processes. A prime source of lead release is automobile discharge [7]. Other metals such as copper, zinc, and arsenic [8] are obtained from the smelting process. Burning of fossil fuels is the main source of mercury, tin, and selenium, while the use of pesticides is a source of arsenic production [9].

A crucial category of emerging contaminants is the dyes whose production is also directly related to the industrial processes. The major sectors employing dyes include the textile industries for the coloring of fabrics, staining of biological and biochemical substances, food industries using dyes for enhancing the texture of their food products, cosmetics, leather goods, paint, and pigments [10, 11]. Another breakthrough in the industrial sector is the pharmaceutical industry production of an enormous stock of medicines for various conditions [12]. However, in contrast to the health benefits obtained through the production of such medicines, the disposal of various antibiotics into the environment is putting the lives of humans at risk. Additionally, expired medicines are also disposed of, which is causing contamination and ill effects [13]. The same logic applies to the use of pesticides and surfactants, which, once used to the degree of demand, are left unchecked in the environment, causing toxic effects. The different types of pesticides, herbicides, and insecticides sprayed on crops for better yields typically do not completely vanish, thus making their way to the food chain and causing detrimental health issues [14–16]. These pollutants have recently been categorized as emerging contaminants that can cause devastating ecological effects directly influencing human health [17]. Humans are the primary targets for such contaminants, directly by water consumption or indirectly through the food chain. The environmental deterioration caused by such toxic contaminants ultimately causes health-related issues for individuals. These contaminants are known to be carcinogenic and mutagenic substances in most cases [18].

Heavy metal pollutants are very harmful. For example, cadmium, which is a human carcinogen, causes destructive effects on the lungs, possible kidney diseases, and bone fragility [19, 20]. Chromium in the form of chromium(III) is an essential nutrient, while chromium (VI) is highly toxic. The common health-related issues associated with chromium include asthma, cough, shortness of breath, or wheezing [21]. Lead has proved to be the cause of various ailments including damage to brain and kidney cells ultimately leading to death [22]. Another harmful metal is mercury which may permanently damage critical body organs. This causes malfunctioning of the brain, resulting in irritability, behavioral changes, tremors, and reduced vision or hearing [23, 24].

Dyes are another form of toxic contaminants causing harmful effects due to their excessive usage. The toxicity of the dye is associated with the azo group present in its structure, making it a complex system. The azo group is defined as possessing a central nitrogen–nitrogen double bond and is hence electron-deficient [25]. The presence of such azoic dyes in wastewater is highly visible, affecting the transparency of water and causing aesthetic disadvantages. The more important aspect of the presence of such compounds in wastewater is their harmful effects on human health, causing several disorders including allergies, cramps, kidney failure, liver damage, and genetic mutations [26, 27]. Pharmaceuticals come under the category of biologically active compounds that have been developed for disease control in living organisms [28].

Apart from the beneficial effects of these pharmaceutical compounds, their biological activity may also affect non-target organisms in adverse ways, thereby harming the ecosystem function and associated ecosystem services. Also, unsupervised disposal of unused or expired pharmaceuticals into the environment may have serious health effects. The most common health-related issues caused by pharmaceutical contaminants include hormonal disruptions, infertility, and colorectal tumors [29–32].

Other contaminants include phosphates and nitrates, which also have serious health and environmental impacts. Although nitrate itself is nontoxic, its conversion into nitrite causes a condition called methemoglobinemia by interfering with the ability of hemoglobin to take up O_2_, which causes cancers of the digestive tract [33–35]. The environmental effects of phosphates and nitrates in wastewater contribute to the phenomenon of eutrophication, leading to harmful algal blooms [36–38]. These issues demand the development of safety measures against these harmful contaminants. Thus, the need for contaminant removal has become critical. Researchers have been focusing on developing such strategies for the removal of toxic contaminants with minimal labor and cost while achieving effective results.

**Scheme 1:**
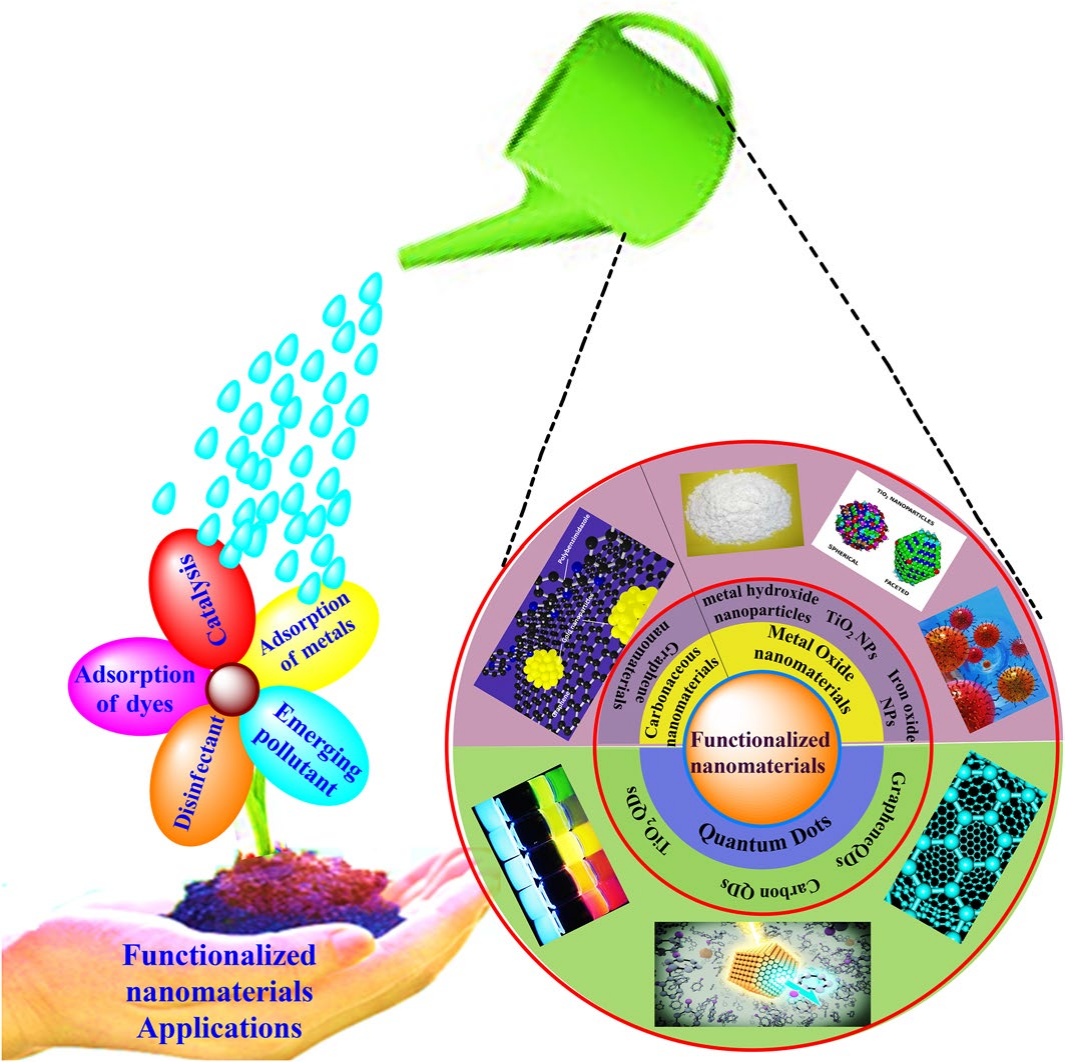
Possible applications of covalent/non-covalent functionalized nanomaterials

Different techniques applied to abolish pollutants in water bodies include ion-exchange [39], reverse osmosis [40], chemical precipitation [41], membrane filtration [42], coagulation and flocculation [43], irradiation [44], electrochemical treatment techniques [45], and advanced oxidation processes [46, 47]. These strategies are being exploited extensively in the context of toxic contaminant removal against wastewater bodies, but their activity is limited for one reason or another. The applications of these strategies may be affected by numerous factors, for example, handling productivity, operational strategy, vitality necessities, and monetary advantage [48–54]. The quest for the development of such a technique offering better removal of contaminants has turned the attention of researchers towards sorptive and photocatalytic techniques. Both sorption [55] and photocatalytic techniques [56–58] have enabled the complete removal of contaminants, and many studies in the literature confirm the contaminant removal efficiency of these techniques (Scheme 1).

The classical materials which have been used to date for the sorption and photocatalysis of contaminants are now being replaced by nanomaterials (NMs) due to their innovative and efficient approach. The review presented herein compiles the most recent and innovative utilization of functionalized nanomaterials (FNMs) in environmental remediation, highlighting the importance of FNMs in the present era.

## Functionalized Nanomaterials, an Innovative Approach Towards Environment Remediation

**Fig. 1:**
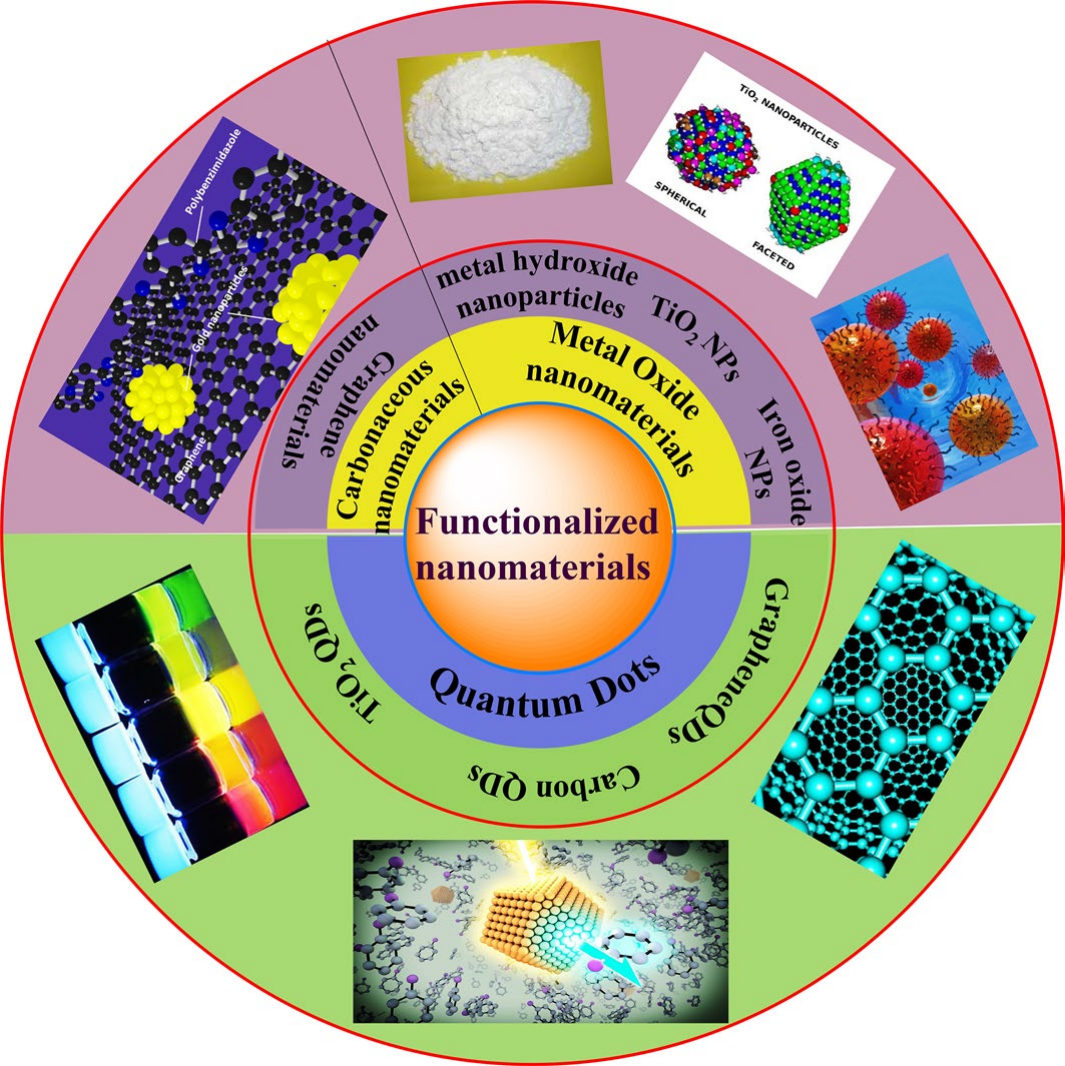
Covalent/non-covalent functionalized nanomaterials

Recent advancements in nanotechnology have provided good alternatives for upgrading wastewater treatment processes. The robustness of nanoscience has taken over the globe due to its remarkable features relying mainly upon the particle size and surface-to-volume ratios [59]. NMs, which feature nanoscale dimensions (less than 100 nm), have garnered considerable attention because of their extraordinary magnetic, synergist, and electronic properties [60, 61]. Because of these unique properties, many efforts have focused on the potential use of NMs for environmental remediation [62]. In this way, NMs have revolutionized the environmental remediation and sensing fields, offering better efficiency in wastewater contamination. Figure 1 outlines some of the well-known FNMs.

**Fig. 2:**
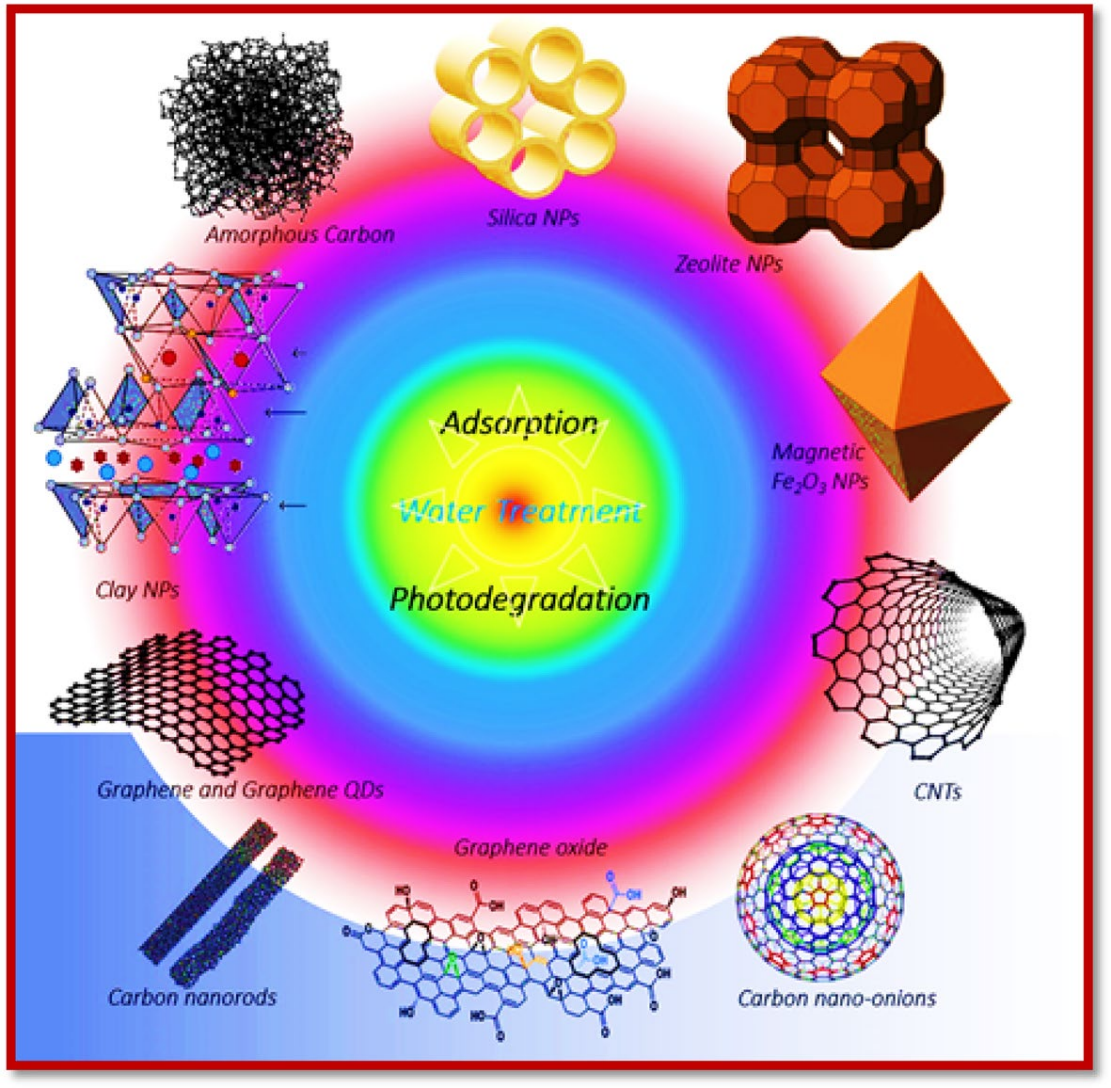
Illustration of photodegradation and adsorption process for wastewater treatment. Reproduced with permission [74] Copyright 2020, Elsevier

NMs of different shapes/morphology—for example, nanoparticles [63], nanotubes [64], nanowires [65], and nanofibers [66]—provide a choice for environmental restoration mainly through the removal of toxic contaminants [67]. NMs play a huge role in contaminant removal due to the high surface area (surface-to-volume ratio) and related high reactivity [68, 69]. As NMs additionally offer extraordinary dependability, effectiveness, and size-dependent optical features, their utility in multiple fields, including sensing, [70], drug delivery frameworks [71], catalysis [72], gas/energy storage [73], and sorption [74–77], is huge (Fig. 2). As a result, NMs have proved to be an important aspect of environmental remediation strategies.

Many NMs have been utilized in the context of contaminant removal from wastewater bodies. In some instances, the activity of the NMs may be inhibited due to various factors including their insolubility in physiological buffers, instability, and low efficiency, recovery, and recyclability [78, 79]. These issues can be resolved through functionalization of NM van der Waals forces, π–π stacking, charge transfer, and/or hydrophobic interactions of the NMs with functionalizing agents [80]. Important advantages associated with the functionalization of NMs include corrosion control, molecular electronic junctions, and complexing layers for the removal of contaminants [81]. Hence, FNMs have proved to be an excellent choice for contaminant removal based on their properties. The most common limitation of NMs is their strong van der Waals forces; they tend to aggregate, thereby reducing the surface availability for better functioning.

Also, the recovery of inorganic NMs is a difficult task that limits the usage and recyclability of the sorbents or photocatalysts [82]. Stability and high porosity are important factors that need to be considered to improve efficiency. To meet the stated standards and achieve better dispersion of NMs, they are often further functionalized to improve their activity. Fabrication or functionalization offers additional sites for the removal of contaminants, thereby enhancing their efficiency. Functionalization of NMs improves their coupling ability towards the analyte, hence enhancing their activity as sorbents or photocatalysts. Such advancement is ideal for the removal of contamination at an ultra-efficient and robust level [83, 84]. Furthermore, functionalized fluorescent NMs tend to have utility for various applications because of their permeability, huge surface area, high stacking limit, and explicit association against toxins such as lead (Pb), cadmium (Cd), copper (Cu), and mercury (Hg) [85]. Also, functionalization of NMs may significantly strengthen them in aqueous solutions by wrapping them superficially with stabilizing agents, for example, biomolecules, surfactants (cationic/anionic), or natural particles [86, 87].

Functionalized magnetic NMs, comprising both natural and inorganic components, have recently been recognized as highly promising agents for different applications, specifically for serving as sorbents or photocatalysts for the removal of contaminants from wastewater bodies. Due to their superparamagnetic properties, huge explicit surface area, and specific sorption limit, this exceptional class of NMs displays a phenomenal capacity for separating and enhancing various analytes of interest [88].

**Fig. 3:**
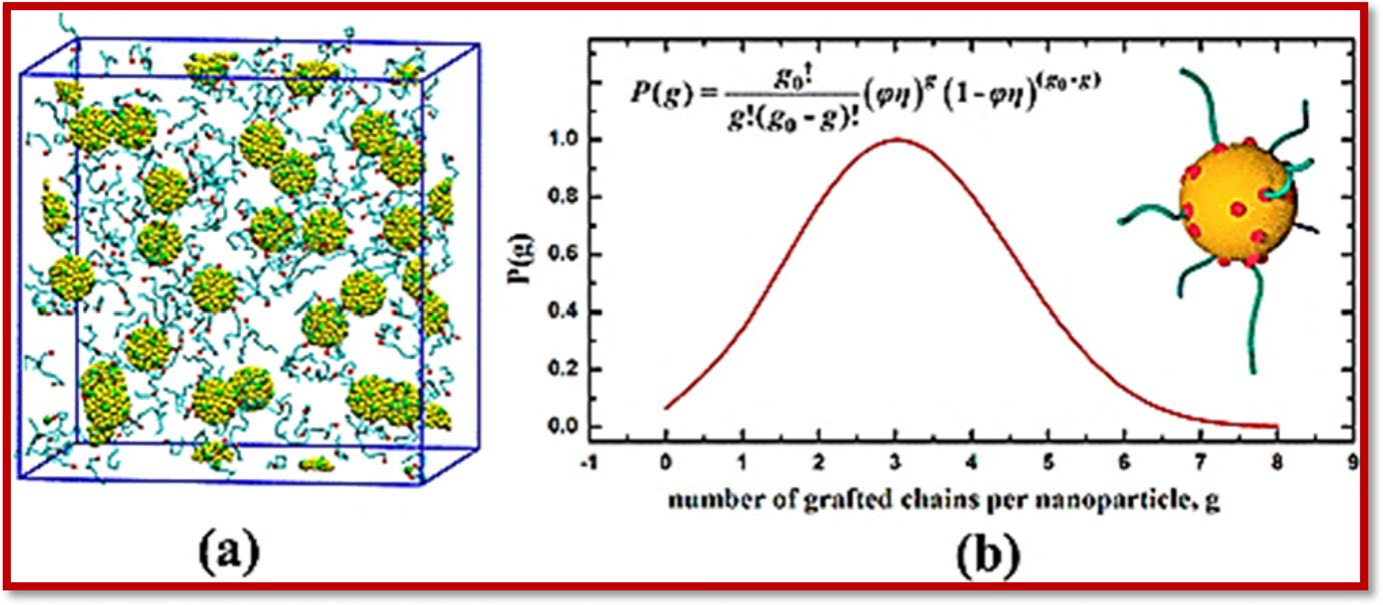
Illustration of the grafting-to reaction. Reproduced with permission [90] Copyright 2018, American Chemical Society

Previously, smaller molecules were used for the functionalization of the NMs, which have now been replaced by large polymeric and biopolymeric molecules [89]. The most commonly utilized techniques for polymer and biopolymer functionalization of NMs include the “grafting-to” and “grafting-from” approaches [90] (Fig. 3). The grafting-from approach allows high-molecular-weight chains of the polymers to be grafted onto the surface of the NMs, minimizing the chances of steric hindrance and resulting in higher-molecular-weight polymer-functionalized NMs. The whole process is completed in three steps: first, the suitable functional group approaches the material, then a covalent interaction is created with the initiator element, and finally the grafting of the polymer onto the surface of the NMs occurs through any of the available techniques.

**Fig. 4:**
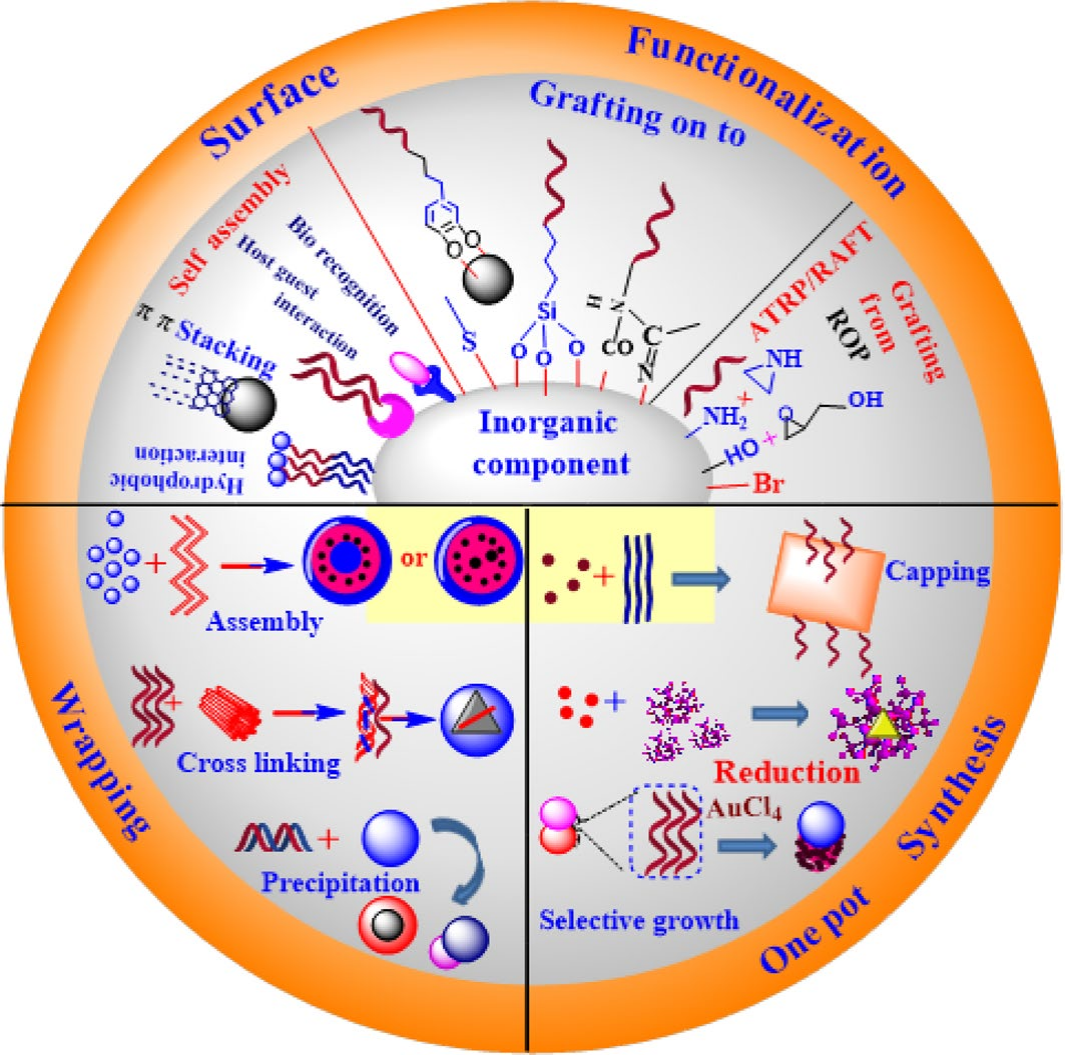
Different techniques used for the synthesis and functionalization of nanomaterials

The techniques used for polymerization onto the NM surface through grafting-from polymerization include atom transfer radical polymerization (ATRP) [91, 92], reversible addition-fragmentation chain-transfer polymerization (RAFT) [93, 94], plasma and UV/O_3_ (UVO)-induced grafting [95, 96], distillation–precipitation–polymerization (DPP) [97, 98], and surface-initiated polymerization (SIP) [99, 100] (Fig. 4). The second concept is the grafting-to approach, which works contrary to the grafting-from approach. According to this, the polymer chains are manufactured first before their linkage to the functional groups of the NMs through amidation, esterification, click chemistry, etc. The grafting-from approach is more frequently used than the grafting onto approach. The most commonly used techniques based on the grafting-to notion include molecular bottle-brush modification [101], Piers–Rubinsztajn (PR) reaction [102], atom transfer radical addition (ATRA) [103], and radical addition. The common feature in these techniques is that they incorporate the polymeric chains with the NMs, introducing additional functionalities inside the NMs, eventually increasing their activity, stability, and efficiency for environmental restoration.

## Trends in the Techniques for the Removal of Contaminants

The functioning of FNMs involves certain pathways depending upon their applications. The NMs that are specifically used for environmental remediation purposes also follow a certain route to remove contaminants. A variety of procedures have been reported to date that make use of the FNMs, but two of these procedures have been highly exploited due to their greater simplicity and efficiency. These methods involve the sorption [104] or photocatalytic [105, 106] pathway for the removal of contaminants. This review will cover the importance of FNMs in environmental restoration with regard to the sorptive and photocatalytic mechanisms.

### Sorptive Removal of Contaminants

**Fig. 5:**
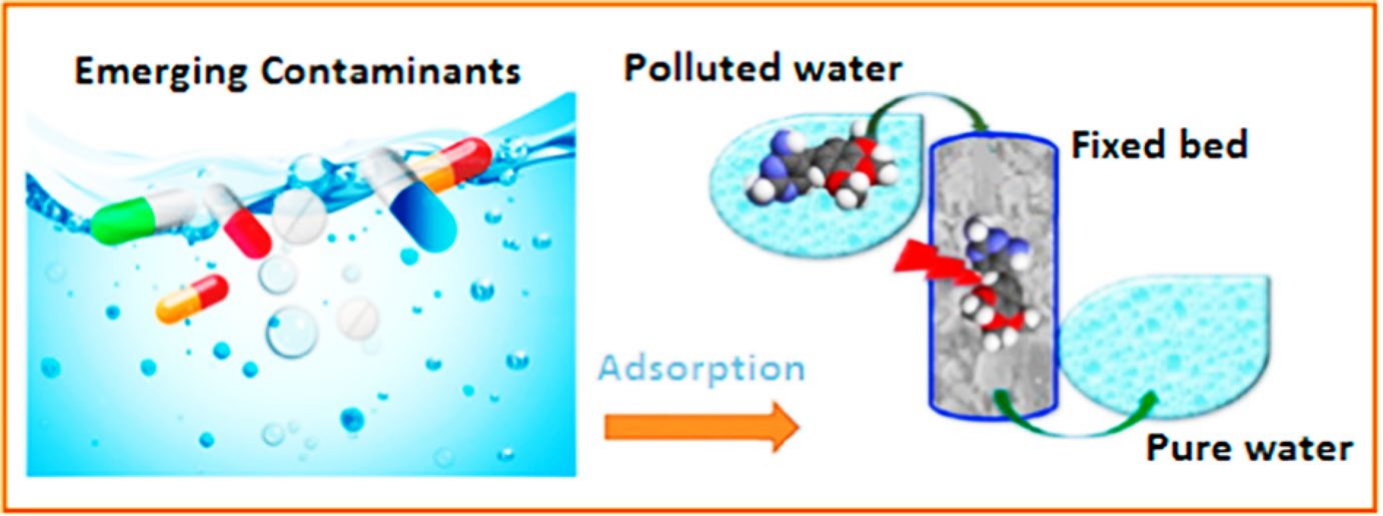
Explanation of the sorption process on a fixed bed for effective removal of pharmaceutical contaminants. Reproduced with permission [109], Copyright 2018, Elsevier

Sorption is preferred over other methods of contaminant removal based on its comparatively straightforward operation, cost-effectiveness, and energy-efficiency. [107]. The constituency of the sorbent material is the prime factor for determining the capacity of the sorption process. Sorption as a common practice for the removal of contaminants, has been widely exploited due to its useful features, particularly its cost-effectiveness, coherence, and feasibility [108]. Because of its one-step process, it can be considered as a simple and facile strategy. The lower costs of the sorption process have made it an appealing choice in underdeveloped and developing countries. Sorption has emerged as a versatile technique for the removal of contaminants and is being widely studied by researchers [109]. Sorption has become a successful one-unit operation for the treatment of industrial waste in recent years (Fig. 5). Although sorption is a commonly utilized technique for the removal of contaminants, its novelty depends on the type of materials used as sorbent. A variety of materials have been introduced that can be used as sorbents at multiple scales [110]. These materials include naturally occurring substances as well as synthetic materials [111].

**Fig. 6:**
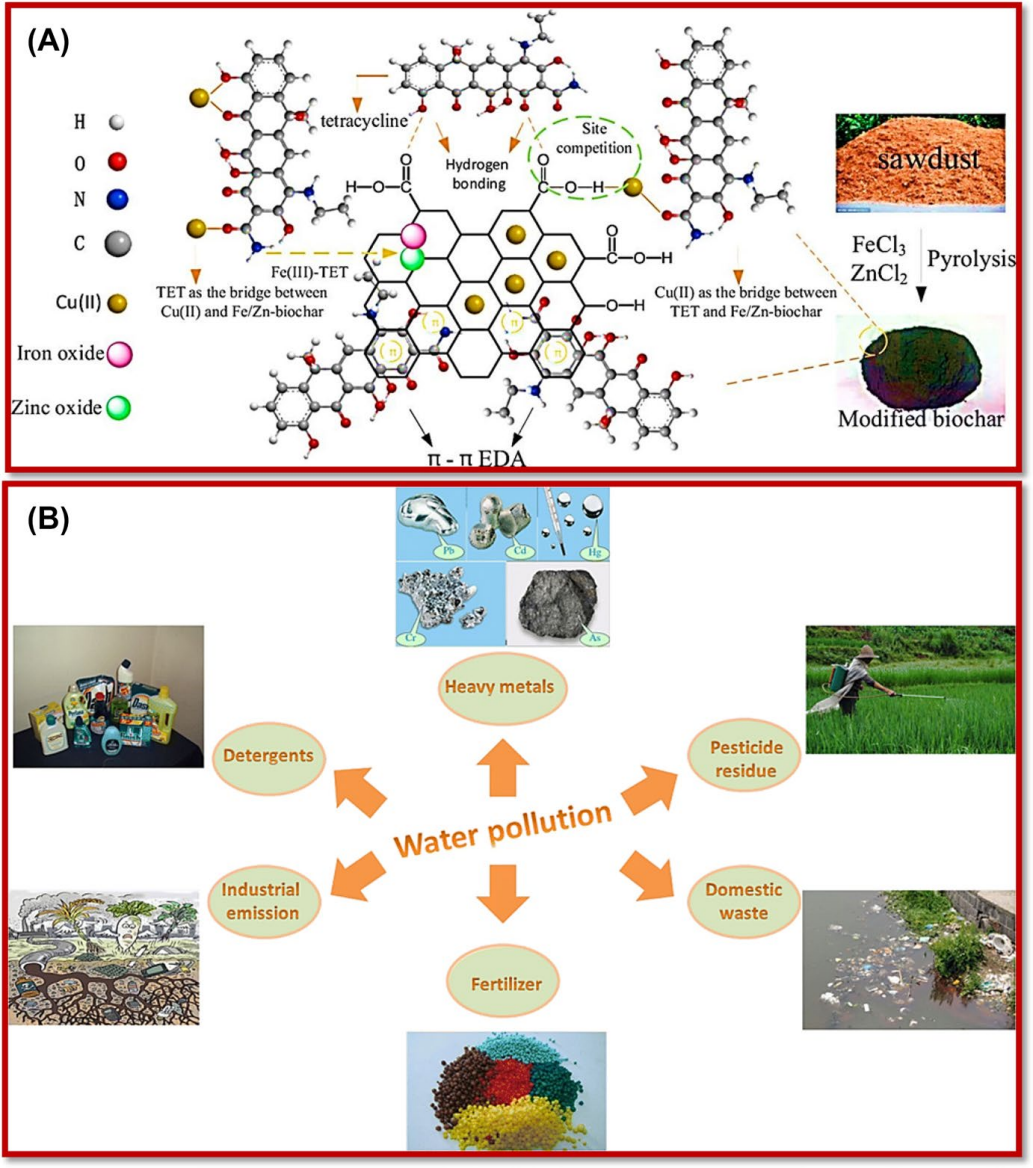
**a** Sawdust-derived biochar and its applications for the removal of copper and tetracycline [118], and **b** explanation of mineral materials for removal of water pollutants released from different sectors. Reproduced with permission [123], Copyright 2017, International Water Association

These materials include low-cost recycled materials such as fruit extracts [112], coconut shells [113], scrap tires [114], fly ash [115–117], sawdust [118], peat moss [119], rice husk [120], red mud [121, 122], minerals [123], blast furnace slag [124] and sludge [125], black liquor lignin [126], waste slurry [127], chitin [128], chitosan [129], and alginate [130]. The synthetic materials used as sorbents include activated carbon [131], zeolites [132], metal oxides/hydroxides [133], metal sulfides [134], and metal selenides [135]. Figure 6 shows some biochar and mineral-derived NMs for effective wastewater treatment.

FNMs have recently proved to be a productive choice for the removal of contaminants. The use of FNMs as sorbents offers more novelty, high efficiency, and simplicity, which increases the interest of researchers in this discipline. A variety of reports are available that use FNMs as sorbents for the removal of contaminants, including thiol-functionalized magnetite nanoparticles [136], carboxyl- and amine-functionalized nanoparticles [137], polyrhodanine-functionalized aluminum oxide [138], TiO_2_ fabricated on mesoporous MCM-4 [139], silica-functionalized magnetic nanoparticles [140], and polymer-functionalized magnetic nanoparticles [141].

### Photocatalytic Removal of Contaminants

Another strategy for the removal of contaminants using FNMs is photocatalysis [142]. A photocatalyst works on the principle of absorbing energy and producing a photon by activation and acceleration of the chemical reaction without undergoing denaturation itself. The photocatalytic phenomenon has been frequently employed by researchers for its useful properties including its efficiency, advanced equipment, and highly efficacious follow-up [143]. The photocatalytic process normally requires an energy source for its initiation, mainly sunlight irradiation. As sunlight is a natural and abundant source of energy, it reduces the energy-related costs for the process. In some instances, UV light is used instead of sunlight as an energy source depending upon the range of the contaminants being studied.

The notion of photocatalysis is to utilize a semiconductor material with a dually functional surface that can act as both cathode and anode performing the activity of a photochemical cell, applicable in various domains including photodegradation [144], photocatalytic CO_2_ reduction [49, 50, 145], photocatalytic synthesis [146], photocatalytic gas-phase oxidation [147], and photocatalytic removal of contaminants [148]. Each of these fields utilizes the concept of photocatalysis at its best. The idea of photocatalysis in the field of contaminant removal eventually leading to the restoration of the environment is growing by leaps and bounds, offering a much broader horizon for its usage [149]. In the case of photocatalysis, the prime factor for choosing the photocatalytic material (photocatalyst) is its semiconductor nature. A semiconductor photocatalyst possesses necessary properties including excellent energy position and bandgap, lower probability of electron–hole recombination, and non-toxicity. The most commonly used materials for photocatalytic purposes include metal oxides [150–152], metal sulfides [153, 154], Fe_2_O_3_ [155], SnO_2_ [156], WO_3_ [157]_,_ metal selenides [158], CuO [159], and Nb_2_O_5_ [160].

The advantages of the properties of FNMs have been used in the fabrication of photocatalysts [161]. Several FNMs are available which have been used as potent photocatalysts for the removal of contaminants including azole-functionalized TiO_2_ [162, 163], Ag-modified metal oxide [164], carboxyl-functionalized metal sulfides [165, 166], and sodium-functionalized quantum dots [167, 168].

## Functionalized Carbon Nanomaterials Robustness in Contaminant Removal

When considering a variety of NMs, carbon-based NMs cannot be overlooked, for they are considered as next-generation materials in multifarious fields [169]. Among the fields utilizing carbon NMs biosensors, drug delivery, biomedical applications, superconductors, and electrically conductive materials are worth mentioning. Because of the useful properties of carbon NMs, for instance, high electrical conductivity and well-defined thermal and mechanical properties, they are used extensively in wastewater treatment [170]. They have proved to be excellent sorbents and photocatalysts on account of their higher surface-to-volume ratio, uniform pore distribution, and highly porous structure. However, despite the very useful properties associated with carbon NMs, their application is sometimes constrained by certain limitations. Carbon NMs have the tendency to agglomerate when they come in contact with the solvent system, which is attributed to their weak van der Waals forces and lower solubility. During synthesis of carbon NMs, some impurities are also formed which may hinder the activity of carbon-based sorbent or photocatalysts. Hence, functionalization of carbon NMs is performed to strengthen their properties and enhance their activity. The functionalized carbon NMs are available in many forms, including carbon nanotubes (CNTs), graphene and its derivatives, and fullerenes. [171].

### Carbon Nanotubes (CNTs)

**Fig. 7:**
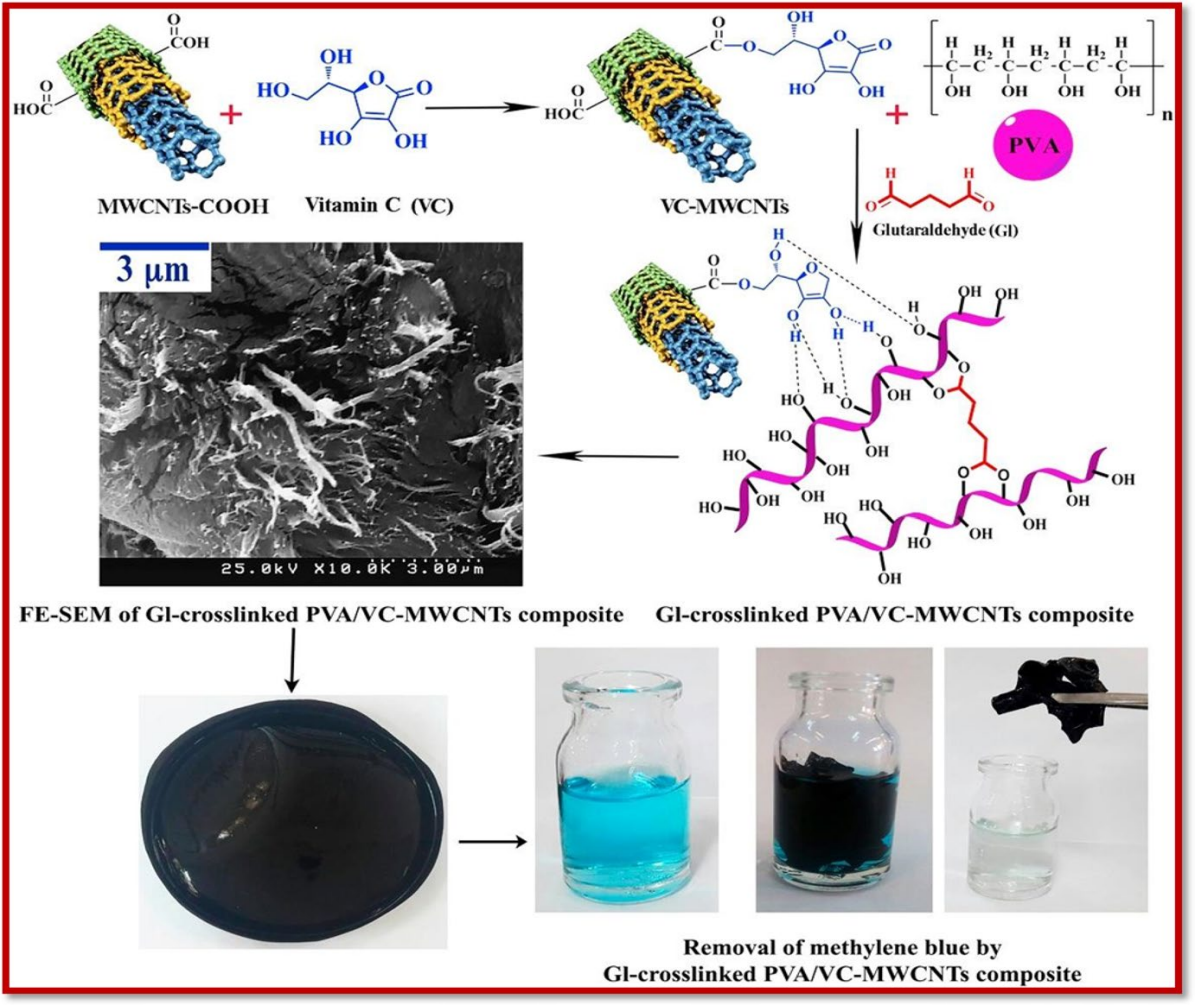
Preparation of vitamin C-MWCNT nanocomposites and their digital pictures and their application for MB dye adsorption. Reproduced with permission [178], Copyright 2019, Elsevier

Carbon nanotubes (CNTs) are the most commonly used form of carbon NMs, and can be pictured as graphene sheets rolled up as tubular cylinders having nanoscale diameters. CNTs are further categorized as single-walled CNTs (SWCNTs), and multi-walled CNTs (MWCNTs). The SWCNTs consist of a single layer of graphene sheet rolled to form a single cylinder, while the MWCNTs consist of multiple graphene sheets arranged as concentric cylindrical sheets [172]. Various methods are available for the synthesis of CNTs including laser ablation, discharge, and chemical vapor deposition. Because of their unique physicochemical properties and structural features, CNTs have been utilized extensively as sorbents and photocatalysts for the removal of various contaminants [173]. The activity of the CNTs can be further enhanced by functionalization with other groups, enhancing their properties such as porosity, hydrophilicity, solubility, and mechanical strength. Alkahlawy et al. [174] performed the photocatalytic degradation of Congo red (CR) dye utilizing MWCNTs modified with zinc oxide and copper oxide nanoparticles. The Zn/CNT photocatalyst possessed excellent activity towards the degradation of CR dye, with degradation efficiency of 97.7% in 70 min under visible light irradiation. The studies showed that the photocatalytic efficiency of Zn/CNTs is directly related to the high dislocation density (*δ*) value of 55.4. This value represents the number of vacancies and defects present in the crystal lattice. The lattice deficiency is the result of the route of synthesis. These intrinsic point defects present in the lattice as atomic impurities, vacancies, and interstices can be detected in doping materials. These defects may function as holes when exposed to active centers, which confirms the dependence of photocatalytic efficiency on the chemical structure rather than texture. The study of the mechanism of photocatalytic activity showed that in a surface defect state, holes/electrons can be trapped, preventing recombination and increasing the oxidation–reduction rate. Structural analysis showed that a number of surface defects were found in the ZnO/CNT sample [175]. These defects mainly consisted of bandgap acceptor states which trap the holes, preventing recombination. Hence, the increased photocatalytic efficiency of the prepared photocatalyst was attributable to the large number of acceptor states caused by the ZnO defects. In addition to the expansion of the light absorption edge of visible light, the acceptor states also cause a delay in the recombination of electron–hole pairs. Hence, the highly defined photocatalytic efficiency can be attributed to the presence of a large number of defects and acceptor states. The ZnO defects act as electron acceptors or hole donors, promoting the position of carrier charges and thus prolonging the separation via trapping at energy levels closer to the conduction band (CB) or valence band (VB), respectively [176]. The photogenerated electrons (e_CB_^−^) may also interact with the electron acceptors, such as oxygen sorbed on the surface of the nanocomposite or dissolved in water, forming radical anions (O_2_^−^ superoxide). The photogenerated holes will also react to form hydroxyl radicals. As result, the highly reactive species produced, OH^**·**^, HO_2_^**·**^, and O_2_^**·**^, will eventually cause the degradation of CR dye. Li et al. [177] explored the sorption ability of functionalized MWCNTs (f-MWCNTs) using crystal violet (CV) and rhodamine B (RB) dye. The sorption of each dye was performed individually and then in a binary system. Individually, the sorption capacity of f-MWCNT for the CR was in the range of 0.57–0.86 mmol/g, while the sorption capacity of RB was 0.75–0.88 mmol/g. The study of individual sorption capacity was related to the physical interaction forces between the f-MWCNT and the dye molecules irrespective of the size, thus resulting in almost equal sorption capacity for both. The mechanistic studies focused on the sorption of both dyes due to the interaction with hydrogen, thus forming associations with COOH or C–H groups of the sorbent molecule [178]. But in the case of a binary system, the sorption capacity of CV was 0.90–1.64 mmol/g, while the sorption capacity for RB was 0.51–0.84 mmol/g. The results clearly showed increased (almost double) sorption of the CV dye, while the sorption of RB was decreased. This trend in sorption capacity in a binary system for both dyes was explained based on synergistic and antagonistic sorption mechanisms. The sorption models applied to the sorption of both dyes in a binary system indicated that the sorption of CV was enhanced in the presence of RB dye, while the opposite was the case for RB dye in the presence of CV dye. The sorption energy also directly affected the sorption process, which seemed to increase in the binary system, hence causing an increase in CV sorption Fig. 7 shows the adsorption of methylene blue (MB) dye on vitamin C-MWCNT nanocomposites.

Shao et al. [179] performed decontamination of polychlorinated biphenyls (PCBs) in aqueous solutions on the surface of MWCNTs grafted with β-cyclodextrin (β-CD) through a plasma technique to enhance the chemical functionality. The maximum sorption capacity achieved by MWCNT-g-CD for 4,4′-DCB (4,4′-dichlorobiphenyl) and 2,3,3′-TCB (2,3,3′-trichlorobiphenyl) was 261 mg/g and 235 mg/g, respectively. A comparison of the sorption capacity of the MWCNTs and MWCNT-g-CD was also performed to evaluate 4,4′-DCB (4,4′-dichlorobiphenyl) and 2,3,3′-TCB (2,3,3′-trichlorobiphenyl) sorption efficiency, and a greater percentage was sorbed by the grafted MWCNT-g-CD than the non-FNMs. This higher sorption capacity possessed by the MWCNT-g-CD depends on the van der Waals and hydrophobic interactions of the sorbates with the surface of the MWCNT-g-CD. The sorption process is a surface phenomenon and is related to the surface sites available for the sorbate molecules [180]. The interstitial surface and grooves of the sorbent molecule may not be available for the sorption process. The plasma grafting of MWCNTs with β-cyclodextrin provided complex formation with the 4,4′-DCB and 2,3,3′-TCB, which enabled the entrance of the sorbate molecules inside the inclusions of the MWCNT-g-CD, thus enhancing the sorption process.

The functionalized CNTs used for the sorption and photocatalysis of contaminants are shown in Table 1.

**Table 1:** Carbonaceous nanomaterials in wastewater remediation

Nanomaterial	Contaminant	Mechanism	Sorption/catalytic capacity	Studied conditions			Cycles	References
				pH	Time	Temp °C		
(MWCNT)	Co^2+^	Sorption	78.94 mg/g	10	38.6			[181]
γ-alumina			75.78 mg/g	10	35.5			
(MWCNT)	Cr(III)	Sorption	8.4 mg/g		30	20		[182]
Magnetic tubular carbon nanofibers (MTCFs)	Cu^2+^	Sorption	375.93 mg/g	6	10	25	6	[183]
Lanthanum hydroxide-oxidized multi-walled carbon nanotubes (La(OH)_3_-OxMWCNTs)	p-Nitrophenol	Electrochemical determination	95.62–110.75%	7				[184]
Sulfur-coated magnetic multi-walled carbon nanotubes (S-M-MWCNTs)	Hg(II)	Sorption	> 90%	4–8	180		8	[185]
Carbon nanotubes grafted with polyethylene glycol (CNT/PEG)	Phenol	Sorption	21.23 mg/g	6	30	25		[186]
Magnetic multi-walled carbon nanotubes modified with polyaluminum chloride (M-PAC-MWCNTs)	Humic acid	Sorption	98.57%	7	30	25		[187]
Linear β-cyclodextrin-functionalized multi-walled carbon nanotubes (MWCNTs-CDP)	U(VI)	Sorption	89.54 mg/g	6	180	70		[188]
Amine-modified magnetic, multi-walled carbon nanotubes	Bisphenol-A	Sorption	43.48 mg/g	12	45	30	5	[189]
Fungal mycelium coupled with carbon nanotubes (FM-CNTs)	Cu^2+^	Sorption	342.22 mg/g	5				[190]
Barium chromate and multi-walled carbon nanotubes composite MWCNT/BaCrO_4_)	Evans blue	Photocatalytic degradation		8			4	[191]
Oxidized multi-walled carbon nanotubes (MWCNTs)	Se(VI)	Sorption	1.865 mg/g	7		30		[192]
Multi-walled carbon nanotubes (MWCNTs)	Cu(II)	Sorption	3.19 × 10^–5^ mol/g	5		30		[193]
Activated carbon			2.31 × 10^–4^ mol/g					
Graphene oxide			1.18 × 10^–3^mol/g					
Multi-walled carbon nanotubes (MWCNTs)	Direct blue 53	Sorption	409.4 mg/g	2	240	50	5	[194]
Powdered activated carbon (PAC)			135.2 mg/g					
Functionalized multi-walled carbon nanotubes (f-MWCNTs)	Direct Congo red	Sorption	148 mg/g	3	120			[195]
	Reactive green HE4BD		152 mg/g	5				
	Golden yellow MR		141 mg/g	7				
Multi-walled carbon nanotubes (MWCNTs)	Humic acid	Sorption	31.37 mg/g	4	180			[196]
Nitrogen-functionalized multi-walled carbon nanotubes (MWCNT-ttpy)	Pb^2+^	Sorption	36.23 mg/g	4.5	1440	20		[197]
	Zn^2+^		32.60 mg/g	5.5				
Multi-walled carbon nanotubes/iron oxide composites (MIO–CNTs)	As(V)	Sorption	47.41 mg/g	8		25		[198]
	As(III)		24.05 mg/g					
Multi-walled carbon nanotube/graphene oxide composite combined with polyaniline and doped with para toluene sulfonic acid (*p-*TSA-Pani@GO-CNT)	Cr(IV)	Sorption	142.8 mg/g	2		30		[199]
	Congo red		66.66 mg/g	5				
Nitric acid-modified carbon nanotubes (MCNT-HNO_3_)	Cd(II)	Sorption	26.88 mg/g	5.6	300			[200]
Zero-valent iron nanoparticles immobilized on multi-walled carbon nanotubes (nZVI/MWCNTs)	Direct red 23	Sorption	100%	4	10	40		[201]
Magnetic oxidized multi-walled carbon nanotubes	Rhodamine B	Sorption	33.42 mg/g	6	120	25	5	[202]
Single-walled carbon nanotubes (SWCNTs)	Reactive blue 4	Sorption	567.7 mg/g	2	240	25		[203]
Multi-walled carbon nanotubes (MWCNTs)			502.5 mg/g					
Β-cyclodextrin grafted multi-walled carbon nanotube/iron oxide (CD/MWCNT/iron oxide)	Zn(II)	Sorption	44.43 mg/g	6.5	360	70	6	[204]
Multi-walled carbon nanotubes (MWCNTs)	Triclosan	Sorption	166.8 mg/g	3		15		[205]
Modified magnetic multi-walled carbon nanotubes (MMWCNTs)	Pb^2+^	Sorption	67.25 mg/g	5		25		[206]
	Zn^2+^		3.75 mg/g					
Magnesium oxide anchored carbon nanotubes	Sulfadiazine	Photocatalytic degradation	45 mg/L	11	180		6	[207]
Magnetic carbon nanotube (M-CNT)	Direct red 23	Photocatalytic degradation	100%		60	25		[208]
	Direct red 31							
	Direct Red 81							
Magnetic multi-walled carbon nanotubes/cerium dioxide nanocomposite (MMWCNTs-CeO_2_)	Methylene blue	Photocatalytic degradation	97.5%				5	[209]
Palladium nanocubes supported multi-walled carbon nanotubes (MWCNTs/Pd)	Methyl orange	Photocatalytic degradation	99%		8			[210]
Multi-walled carbon nanotube (MWCNT)	Acid red 14	Photocatalytic degradation	Up to 100%	3	60	25		[211]
	Acid red 18							
Single-walled carbon nanotubes (SWCNTs)	Basic red 46	Sorption	38.35 mg/g	9	80	25		[212]
Carboxylate-functionalized SWCNTs (SWCNT-COOH)			49.45 mg/g					
Graphene			30.52 mg/g		90			
Graphene oxide			55.57 mg/g					
Multi-walled carbon nanotubes	Alizarin red S	Sorption	161.2 mg/g	1	30	30		[213]
	Morin		26.2 mg/g					
Acid-oxidized multi-walled carbon nanotubes (CNTs)	Mn(VII)	Sorption	100%		240			[214]
	Cr(VI)				180			
	Azo dyes (toluidine blue, methylene blue, methyl green and bromopyrogallol red)				5			
Multi-walled carbon nanotubes (MWCNTs)	Eriochrome cyanine R	Sorption	95.2 mg/g	2	10	25		[215]
Activated carbon (AC)			40.6 mg/g	3	41			
Multi-walled carbon nanotubes (MWCNTs)	Arsenazo III	Sorption	30.58 mg/g	1	15	25		[216]
Activated carbon (AC)	Arsenazo III		10.2 mg/g	1	50			
	Methyl red		46.29 mg/g	7	15			
Multi-walled carbon nanotubes (MWCNTs)	Reactive red M-2BE	Sorption	335.7 mg/g	2		25	4	[217]
Powdered activated carbon (PAC)			260.7 mg/g					
Single-walled carbon nanotubes (SWCNTs)	Reactive blue 29	Sorption	66.3%	5	240			[218]
Alkali-activated carbon nanotubes CNTs-A	Methyl orange	Sorption	149 mg/g	2				[219]
	Methylene blue		399 mg/g					
Single-walled carbon nanotubes (SWCNTs)	Reactive red 120	Sorption	426.49 mg/g	5	180	25		[220]
Acidified ammonium persulfate-treated single-walled carbon nanotubes (t-SWNTs)	Bisphenol A	Sorption	8 mg/g	7.5				[221]
	17β-Estradiol		27.4 mg/g					
Carbon nanotubes (CNTs)	Cephalexin	Sorption	Up to 97.6%	5				[222]
Multi-walled carbon nanotubes (MWCNTs)	Tetracycline	Sorption	269.54 mg/g	4.8	20	20		[223]
Multi-walled carbon nanotubes (MWCNTs)	Atenolol, caffeine, diclofenac and isoproturon	Sorption		3–9				[224]
Activated carbon (AC)								
Carbon nanofibers (CNFs)								
Single-walled carbon nanotubes (SWCNTs)	Ethidium bromide	Sorption	36.10%		3	25		[225]
Carboxylate-functionalized single-walled carbon nanotubes (SWCNTs-COOH)			38.42%		6			
MWCNT-stabilized Pd/Fe nanocomposites	2,4-Dichlorophenol	Sorption	Up to 95%	4	120	30	3	[226]
Cyclodextrin-phosphorylated MWCNT polymer	Co	Sorption	67.7%					[227]
	4-Chlorophenol		87%					
KOH-activated carbon nanotubes (CNTs-KOH)	Toluene	Sorption	87.12 mg/g	6		20		[228]
	Ethylbenzene		322.05 mg/g					
	m-xylene		247.83 mg/g					
Ammonia-functionalized micron-sized activated carbon fibers (ACF)	Phenol	Sorption	200 mg/g			38		[229]
	Pb^2+^		More					
Carbon nanofibers/activated carbon multiscale web (ACF/CNF)	Pb^2+^		Less					
	Phenol		150 mg/g					
Carbon nanofibers	Ciprofloxacin	Sorption	0.68 ± 0.04 mmol/g	2–6				[230]
	Bisphenol		4.82 ± 0.22 mmol/g					
	2-chlorophenol		6.18 ± 1.64 mmol/g					
Carbon nanotubes (CNTs)	Cd^2+^	Sorption	2.02 mg/g	7	120			[231]
Carbon nanofibers (CNFs)			1.22 mg/g					
Activated carbon (AC)			1.98 mg/g					
Fly ash (FA)			1.58 mf/g					
Multi-walled carbon nanotubes (MWCNTs)	Tetracycline	Sorption	253.38 mg/g	5–7	120	20		[232]
Carbon nanotubes (SWCNTs)	Amoxicillin	Sorption	99.1%		45	50		[233]
Multi-walled carbon nanotubes (MWCNTs)	Diuron	Sorption	29.75 mg/g	7		25		[234]
Multi-walled carbon nanotubes (MWCNTs)	Perflourooctane sulfonate	Sorption	0.71 mmol/g					[235]
	Perfluorooctanoic acid		0.32 mmol/g					
	Perfluorooctanesulfonamide		1.37 mmol/g					
	2,4-Dichlorophenoxyacetic acid		0.79 mmol/g					
	4-*n*-Nonylphenol (4-nanoparticle)		0.83 mmol/g					
Multi-walled carbon nanotubes (MWCNTs)	Amoxicillin	Sorption	22.9 mg/g	4.6	120			[236]
Activated carbon with different oxy-acids pf phosphorus AC-H_3_PO_4_	Trimethoprim	Sorption	1.1478	10				[237]
AC-H_4_P_2_O_7_			1.1896					
AC-HPO_3_			0.4098					
AC-H_3_PO_3_			0.4084					
Carbon nanofibers decorated with iron nanoparticles, porous carbon microbeads	Cr(VI)	Sorption	41 mg/g	2	1200		5	[238]

### Graphene and Its Derivatives

As an important topic in any in-depth discussion of carbon-based NMs, graphene is considered to be the simplest form of carbon and is also the thinnest material known. Graphene is unquestionably an extraordinary material that has been used extensively in various fields [239]. The structural assay of graphene shows that it consists of a two-dimensional single-layer sheet of carbon atoms that are arranged into an sp^2^-bonded honeycomb-like lattice structure. The properties associated with graphene, including thermal stability, larger surface area, mechanical strength, electrical conductivity, and flexibility, make it a highly promising candidates for use in wastewater treatment processes [240, 241]. The specific properties of high surface area, delocalized π-electron system, and abundantly present active sites indicates excellent sorption capacity inhibited by the graphene-based NMs. Not only has pristine graphene been used massively in the removal of contaminants, but the derivatives of graphene have also been utilized [242]. Many reports are available on the utilization of graphene oxide, reduced graphene oxide, graphene platelets, and graphene-based composites as potent sorbents as well as photocatalysts. The properties of graphene NMs can be further enhanced by performing the functionalization procedure through groups such as thiol moieties or carboxylic groups [243].

Zamani and Salem [244] studied the photocatalytic behavior of graphene oxide sheets coupled with carbon nanotubes. In the first step, functionalization of carbon nanotubes was performed using acid reflux conditions, and they were then coupled with graphene oxide and decorated with anatase using the sol–gel method [245]. The obtained nanocomposite showed 96.5% efficiency for the degradation of MB dye. The hybrid nanocomposite facilitated the degradation of the contaminants under solar irradiation by providing numerous active sites for the photoreactions. The hybrid nanocomposite had reduced bandgap energy of 2.2 eV, facilitating electron/hole pair separation. A detailed analysis of the photocatalytic degradation of the MB dye shows that the connection of graphene oxide with anatase nanoparticles further improves the photocatalytic efficiency by prolonging the electron recombination. It is observed that the high graphene oxide loading may cause agglomeration which affects the electron transfer [246]. Hence, the incorporation of CNTs creates spaces between the sheets of GO, facilitating a connection between carbon-based materials and anatase particles. This improved connection between the carbon-based materials and TiO_2_ then reduces the bandgap and enhances the photocatalytic activity. When the nanocomposite is exposed to solar light irradiation, the es^−^ are excited from the VB to the CB of TiO_2_. When a satisfactory connection is formed between anatase and the CNTs or GO, the es^−^ can be easily transferred to both parts, causing electron–hole pair separation, generating highly active radicals for the degradation of dyes [247]. The MB degradation occurs through the following equations:

$$\mathrm{Nanocomposite }+h\vartheta \to {\mathrm{e}}^{-} + {h}^{+} +\mathrm{Nanocomposite}$$

$${h}^{+} + {\mathrm{H}}_{2}\mathrm{O }\to 2{\mathrm{OH}}^{\bullet}$$

$${h}^{+}+{\mathrm{OH}}^{-}\to {\mathrm{OH}}^{\bullet }$$

$$\mathrm{MB }+ {h}^{+ }\to {\mathrm{CO}}_{2}+{\mathrm{H}}_{2}\mathrm{O}$$

$$\mathrm{MB }+{\mathrm{OH}}^{\bullet }\to {\mathrm{CO}}_{2}+{\mathrm{H}}_{2}\mathrm{O}$$

Wang et al. [248] exploited the efficiency of a novel biosorbent based on graphene oxide modified with persimmon tannin (PT-GO) fabricated with glutaraldehyde cross-linking, for the removal of MB dye. The PT provides a large number of active sites due to the presence of phenolic hydroxyl groups, while GO has abundant hydrophilic groups and also provides a large specific surface area. The modification of GO with PT therefore enhances its sorption capacity by providing stability and additional functionality. The highest sorption capacity obtained was 256.58 mg/g at optimal conditions of pH 8 and temperature of 323 K. The sorbent is rich in phenolic hydroxyl groups due to the presence of persimmon tannin and numerous hydrophilic groups associated with the graphene oxide portion of the sorbent. The mechanistic pathway for the sorption was attributed to electrostatic interactions, redox reactions, and π–π interactions. The analysis of the sorption process shows that the phenolic-hydroxyl groups of the biosorbent adhere to the cationic MB dye through electrostatic interactions. The second step involves the sorption process through a redox reaction between the sorbent and the dye, while a π–π interaction is created between the benzene rings of the dye and the biosorbent.

**Fig. 8:**
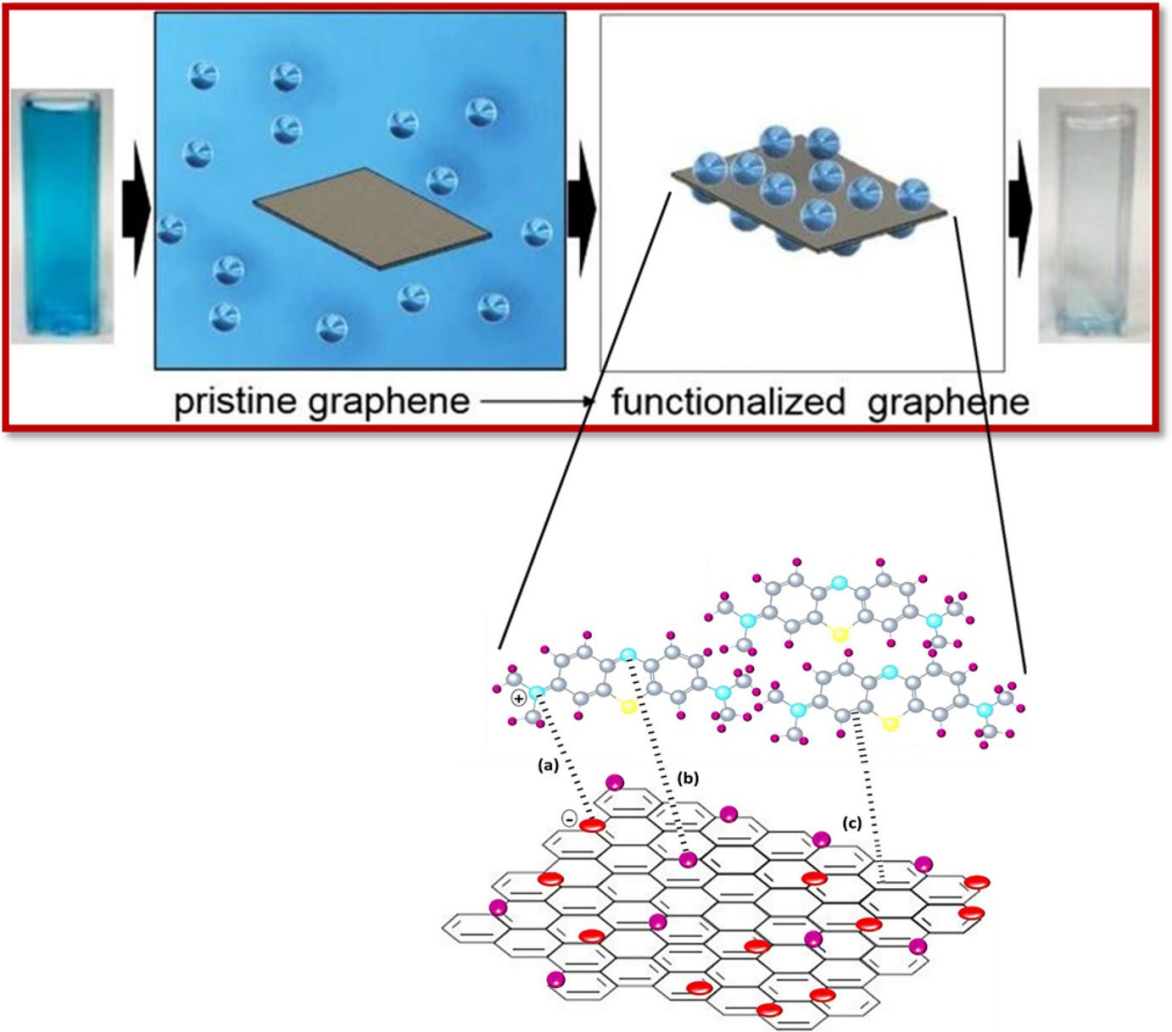
Schematic presentation of the MB adsorption on the f-GNP1 surface. Reproduced with permission [249], Copyright 2019, Springer Nature

Firdaus et al. [249] functionalized graphene nano-platelets (GNPs) with oxygen-containing functional groups (acid oxidation). The functionalization of GNPs was performed using a 1:1 mixture of H_2_SO_4_ and HNO_3_. The functionalized GNPs showed higher sorption capacity (225 mg/g) for MB dye from an aqueous solution (Fig. 8). The higher sorption capacity of the functionalized GNPs was attributed to the enhanced surface area, pore size, and pore volume of the material due to the functionalization of the GNPs. Also, the functionalized GNPs showed stable dispersion in aqueous solution and better hydrophilicity. These properties induced by functionalization enhanced the sorptive capacity of the material towards contaminants.

Karimi-Maleh et al. [250] also studied the preparation of a magnetic nanocomposite sorbent based on reduced graphene oxide (rGO–Fe_3_O_4_) and used it for the removal of phenazopyridine, an azo dye having pharmaceutical attributes, and exhibited sorption capacity of 14.06 mg/g at the optimized conditions. The sorption of the contaminant over the surface of the sorbent was attributed to the presence of hydronium ions (H_3_O^+^) in the spaces between the graphene layers in the acidic conditions, leading to better sorption chances. The aromatic ring of the prepared sorbent and the amino group of the sorbate tend to interact, hence leading to better sorption capacity (Table 2).

**Table 2:** Graphene-based nanomaterial in wastewater remediation

Nanomaterial	Contaminant	Mechanism	Sorption/catalytic capacity	Conditions studied			Cycles	References
				pH	Time	Temp °C		
Graphene oxide	Metformin	Sorption	122.61 mg/g	6.2	180	20	6	[251]
Reduced graphene oxide nanosheets doped with Cu	Methylene blue	Photocatalytic degradation	91%		80			[252]
Nickel(II) hydroxide (Ni(OH)_2_)-decorated graphene oxide (GO) and modified titania (TiO_2_) nanocomposite (Ni(OH)_2_/GO/TiO_2_)	2-Chlorophenol	Photocatalytic degradation	80%	6	240			[253]
Iron oxide/graphene oxide nanohybrid	2,4-Dichlorophenol	Sorption	96.5%					[254]
Reduced graphene oxide-CuO/ZnO (rGO-CuO/ZnO)	4-Nitroaniline	Catalytic degradation	98%		10		4	[255]
Poly(2-hydroxyethyl methacrylate) grafted graphene oxide	Cd	Sorption	89%	7	30	25		[256]
Polyaniline-functionalized magnetic graphene oxide composites (PANI-g-MGOs)	Cd(II)	Sorption	7.63 × 10^−4^	7		25		[257]
	Phenol		2.87 × 10^−3^					
Surface-tailored graphene oxide paper	Methylene blue	Sorption	311 mg/g					[258]
	Methyl orange		340 mg/g					
Silver nanoparticles immobilized on graphene oxide nanosheets	Methylene blue	Sorption	633 mg/g	9	90	27	4	[259]
Magnetic graphene oxide (Fe_3_O_4_/SiO_2_-GO)	Cd(II)	Sorption	128.2 mg/g	5	40	25	12	[260]
	Pb(II)		385.1 mg/g					
Silver incorporated reduced graphene oxide sheets (Ag@rGO)	Methylene blue	Photocatalytic degradation	100%		120			[261]
Magnetic graphene oxide composite (CoFe_2_O_4_/GO)	Methylene blue	Sorption	355.9 mg/g		400		4	[262]
	Rhodamine B		284.9 mg/g					
	Methyl orange		53 mg/g					
Magnetic graphene oxide-titanate composites (MGO@TNs)	Pb(II)	Sorption	322.7 mg/g	5	180		6	[263]
Magnetic porous reduced graphene oxide (MPrGO)	Triclosan	Sorption	1105.8 mg/g	3			5	[264]
Yttrium-immobilized-graphene oxide-alginate hydrogel (Y-GO-SA)	As(V)	Sorption	273.39 mg/g	5		20	4	[265]
	Tetracycline		477.9 mg/g					
Graphene oxide	Trichloromethane	Photocatalytic degradation	99.6%		5			[266]
Graphene oxide/aminated lignin aerogels (GALA)	Malachite green	Sorption	113.5 mg/g	8	600	25		[267]
Polyethersulfone-functionalized GO (PES-fGO)	Fe^2+^	Sorption	97.1%					[268]
	Zn^2+^		95.3%					
	Cd^2+^		92.7%					
	Cr^6+^		99.9%					
Amine-graphene oxide composite (pssN-GO)	Cr(VI)	Sorption	260.74 mg/g	3				[269]
Graphene oxide with lanthanum substituted manganese ferrites (LMFx%@GO)	Perfluorooctanoic acid	Sorption	1.61 mg/g	3	1440	25		[270]
Graphene-based hydrogel (Fe_3_O_4_/RGO/PAM hydrogel)	Rhodamine B	Photocatalytic degradation	90%	4.5	60		10	[271]
Graphene oxide wrapped magnetite nano-clusters (Fe_3_O_4_@GO)	Methylene blue	Sorption	131.10 mg/g	8	400	30		[272]
	Rhodamine B		34.5 mg/g					
	Methyl orange		39.95 g/g					
Reduced graphene oxide	Methylene blue	Sorption	2.6 g/g					[273]
	Congo red		7.6 g/g					
	Lemon yellow		3.2 g/g					
	Cd^2+^		8.4 g/g					
	Pb^2+^		17.9 g/g					
Graphene-based monolith using magnesium ascorbylphosphate (MAP/GBM)	Bisphenol A	Sorption	324 mg/g	7	2880	25	5	[274]
Biochar supported reduced graphene oxide (RGO-BC)	Pb^2+^	Sorption	26.10 mg/g		1440		4	[275]
	Atrazine		67.55 mg/g					
Sodium bisulfate reduced graphene oxide aerogels (S-rGO)	Tetrabromobisphenol A	Sorption	128.37 mg/g	7		25	5	[276]
Biochar-graphene nanosheets composites	Dimethyl phthalate	Sorption	30.78 mg/g		2880			[277]
	Diethyl Phthalate		23.86 mg/g					
	Dibutyl phthalate		21.98 mg/g					
Graphene oxide nanosheets	17β-Estradiol (E2)	Sorption	149.4 mg/g	7	480	25	5	[278]
Graphene oxide metal–organic framework nanocomposite (GO-MOF)	Methylene blue	Sorption	97%		15		4	[279]
Cadmium sulfide reduced graphene oxide (CdS-RGO)	Rhodamine B	Sorption	97.2%				4	[280]
	Acid chrome blue K		65.7%					
Nickel ferrite-reduced graphene oxide (NFRGO)	Pb(II)	Sorption	99%					[281]
Graphene oxide/silver nanocomposite (GO-Ag)	Malachite green	Sorption	143 mg/g		25	30	4	[282]
	Ethyl violet		72 mg/g					
Graphene oxide-polydopamine-(β-cyclodextrin) (GPC)	Pb(II)	Sorption	101.6 mg/g	6	160		5	[283]
	Methylene blue		99.2%					
Silver/reduced graphene oxide nanocomposite (Ag/rGO)	Methyl orange	Photocatalytic degradation	90%	5.5	45		3	[284]
Graphene oxide/gold nanocomposite (GO-Ag)	Malachite green	Sorption	1000 mg/g			25	4	[285]
	Ethyl violet		13.3 mg/g					
Silica-magnetic nanoparticle-decorated graphene oxide (GO-MNPs-SiO_2_)	Naproxen	Sorption	31 mg/g	5	60			[286]
Magnetic chitosan-graphene oxide composite (^m^Ç-GO)	Acid red 17	Sorption	79%	6	60	25	5	[287]
	Bromophenol blue		97%	2				
Reduced graphene oxide	Pb(II)	Sorption	96.6%	4.5	120	30	5	[288]
Graphene oxide nanoflakes	Phenolic compound	Sorption	19-30 mg/g		300			[289]
Magnetic chitosan/graphene oxide biosorbent	Cu^2+^	Sorption	217.4 mg/g	8	70		5	[290]
Graphene oxide sheets decorated with polyaniline nanofibers (GO-PANI)	Zn(II)	Sorption	297.62 mg/g	7	20	25	3	[291]
Graphene oxide (GO)	Cu^2+^	Sorption	97%	6	60	25	5	[292]
Functionalized graphene oxide (GO-COOH)			99.4%					
Cobalt oxide nanoparticles incorporated reduced graphene oxide A-rGO/Co_3_O_4_	Rhodamine B	Sorption	102.9 mg/g	12	300	20		[293]
Palladium supported reduced graphene oxide (Pd NPs/RGO)	4-Nitrophenol	Catalytic reduction	97%		1		8	[294]
	Rhodamine B							
	Methylene blue							
Graphene oxide/cellulose hydrogel	Methylene blue	Sorption	100%	7	15	25	3	[295]
	Rhodamine B		90%					
GO/polyethylenimine (PEI) hydrogels	Methylene blue	Sorption	323.9 mg/g		250	25		[296]
	Rhodamine B		114.4 mg/g					
Polyacrylic acid-functionalized magnetic nanoparticles/graphene oxide composite	Methylene blue	Sorption	291 mg/g	7	1440		5	[297]
Poly(amidoamine)-functionalized graphene oxide (PAMAM-GO)	Se(IV)	Sorption	60.9 mg/g	6				[298]
	Se(VI)		77.9 mg/g					
Graphene oxide nanosorbents	Pb(II)	Sorption	100%	8				[299]
	Cr(VI)							
	Ni(II)							
P25/silver orthophosphate/graphene oxide (P25/Ag_3_PO_4_/GO) ternary composite	Rhodamine B	Photocatalytic degradation	95%		60		5	[300]
Graphene oxide/magnesium oxide composite	Methylene blue	Sorption	833 mg/g	11	20			[301]
Polysulfone-graphene oxide-based porous membranes	Ofloxacin, benzophenone-3, rhodamine B, diclofenac and Triton X-100	Sorption	90%		1440		3	[302]
Graphene oxide	Levofloxacin	Sorption	256.6 mg/g					[303]
	Pb^2+^		227.2 mg/g					
Poly(vinyl alcohol)/poly(acrylic acid)/carboxylate graphene oxide nanosheets (PVA/PAA/GO-COOH)	Methylene blue	Sorption	25.91 mg/g		300		10	[304]
	Rhodamine B		6.75 mg/g					
	Congo red		9.62 mg/g					
Reduced graphene oxide	Methylene blue	Sorption	746 mg/g		20			[305]
4-Aminodiphenylamine-modified graphene oxide	Toluene, ethylbenzene, and p-,o-xylene	Sorption	1.5–3.7 mg/g	4	5	23	7	[306]
Dithiocarbamate-functionalized graphene oxide (GO-DTC)	Basic blue 41	Sorption	128.5 mg/g	4.5	60	25	3	[307]]
	Basic red 46		111 mg/g					
Magnetic chitosan-coated graphene oxide (Fe_3_O_4_©-GO)	Methyl violet	Sorption	11.5 mg/g	10	60	25	4	[308]
	Alizarin yellow R		6.7 mg/g	6				
Ag_3_PO_4_/graphene-oxide (Ag_3_PO_4_/GO) composite	Methyl orange	Photocatalytic degradation	86.7%		30			[309]
	Rhodamine B		100%					
Polydopamine-kaoline with reduced graphene oxide (PDA-rGO-kaoline)	Methylene blue	Sorption	39.66 mg/g	7		27	5	[310]

###  Fullerenes

**Fig. 9:**
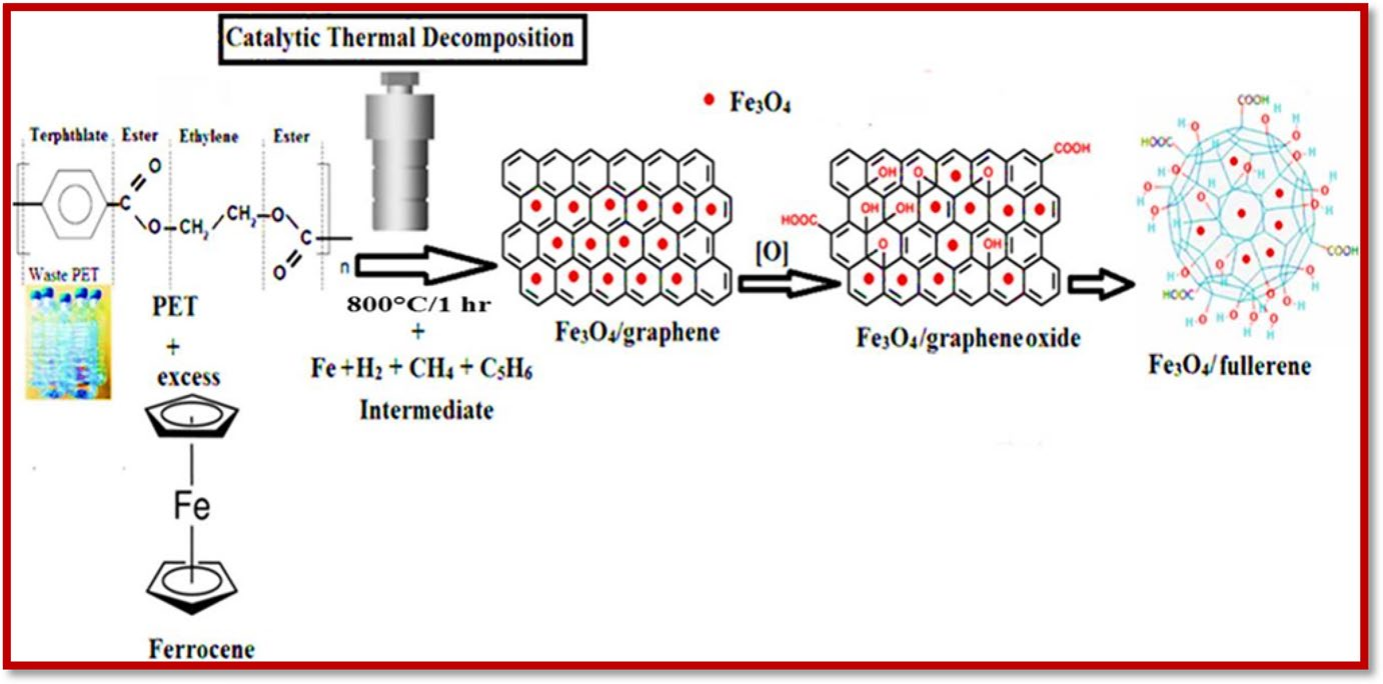
One-step polyethylene terephthalate catalytic dissociation for the synthesis of functionalized magnetic fullerene nanocomposites (FMFN) and different kinds of interactions for the sorption of ciprofloxacin. Reproduced with permission [315], Copyright 2020, Elsevier

Another important form of carbon-based NMs is fullerenes. The fullerenes are composed entirely of carbon atoms, considered as allotropes of carbon. Compared with graphite and diamond, fullerenes are found to be spherical molecules [311, 312]. They show solubility in various organic solvents. The structural elucidation of fullerenes shows that they consist of a carbon cage with a fused ring system mainly comprising hexagons and pentagons. The most generally accessible members of the fullerenes are the C_60_ and C_70_. The most important property of the fullerenes is their high symmetry [313]. Wu et al. [314] performed photocatalytic degradation of RB dye using fullerene-cored star-shaped polyporphyrin-incorporated TiO_2_. The ZnCPP-fullerol@TiO_2_ photocatalyst was prepared by immobilizing fullerene-cored star-shaped polyporphyrin nanospheres, obtained from the esterification of carboxyl porphyrin with fullerols, of the TiO_2_ surface through excess hydroxyl groups of fullerols. The results showed that 94.7% efficiency was obtained for the degradation of RB dye. The good efficiency of the prepared catalyst is attributed to the presence of fullerol, which is a derivative of fullerene and is extensively hydroxylated. The properties of fullerol including its high specific surface area, unique electronic properties, and conjugated aromatic system are the reasons for its use as a charge carrier and photocatalyst. The polyhydroxy groups present on the surface of the fullerene provide high-density active sites. Also, multiple functional groups can be introduced on the surface of fullerenes by the esterification reaction of the hydroxy groups. This synthetic procedure of a molecular-level heterojunction NM accelerates the electron transfer between porphyrin molecules, promoting charge separation. The combined properties of heterogeneous and homogeneous catalysts will enhance photocatalytic degradation efficiency. Elessawy et al. [315] prepared functionalized magnetic fullerene nanocomposites (FMFN) through the catalytic thermal decomposition method. The prepared FMFN was used as a sorbent for the removal of the ciprofloxacin contaminant. FMFN had a high surface area of 336.84 m^2^/g typical of mesoporous and microporous volumes. The saturation magnetization property of FMFN was 7.002 emu/g, confirming its high superparamagnetism. These properties obtained through the functionalization provided a better sorption efficiency (Fig. 9). The highest sorption capacity obtained for the removal of ciprofloxacin was found to be 65 mg/L, which is in complete agreement with the magnificent sorptive ability of FMFN.

Mahdavian [316] analyzed the removal efficiency of heavy metals (nickel and cadmium) by filtration of modified nano-fullerene (C_60_) with tetrahydrofuran. The heavy metal ion sorption was optimized and the results showed that about 91% efficiency was obtained for the removal of ions. A sorption efficiency of 1261 mg/g was obtained for cadmium removal, while sorption capacity of 3704 mg/g was obtained for nickel removal (Table 3).

**Table 3:** Fullerene-based nanomaterial in wastewater remediation

Nanomaterial	Contaminant	Mechanism	Sorption/catalytic capacity (%)	Studied conditions			Cycles	References
				pH	Time (min)	Temp °C		
C_60_-modified ZnAlTi layered double oxide (ZnAlTi-LDO)	Bisphenol A	Photocatalytic degradation	85	7	60	28		[317]
Fullerene (C_60_)/CdS nanocomposite	Rhodamine B	Photocatalytic degradation	97		40		3	[318]
[PdCl_2_, H_2_PtC_l6_·nH_2_O & Y(NO_3_)_3_] Doped TiO_2_	Methylene blue	Photocatalytic degradation						[319]
C_60_	17 CB congeners	Sorption						[320]
Polyhydroxy fullerene (PHF) coated TiO_2_	Procion red MX-5B	Photocatalytic degradation	66–74		360			[321]
Hydroxylated fullerene (fullerenol)	Diethyl phthalate (DEP)	Degradation	100	3.5	60			[322]
Hydroxylated fullerene	Chloramphenicol (CAP)	Degradation	90	3	60			[323]
Fullerenol (polyhydroxyfullerene, PHF)	Acid red 18	Photocatalytic degradation	86.7	< 8	60		4	[324]
[60]Fullerene-functionalized magnetic nanoparticles (Fe_3_O_4_@SiO_2_@C_60_)	Polycyclic aromatic hydrocarbons (PAHs)	Sorption	92.4–106.9	3–12	2–10		10	[325]
Titania nanotubes (TiNTs) functionalized with fullerenes (C_60_)	Isopropanol	Photocatalytic degradation	100		660			[326]
Nanocomposites of TiO_2_ and single fullerene (C_60_) molecule	Methyl orange	Photocatalytic degradation			30			[327]
Rutile-C_60_ composites	Methylene blue	Photocatalytic degradation	100%		240			[328]
Fullerene modified C_3_N_4_ (C_60_/C_3_N_4_) composites	Rhodamine B	Photocatalytic degradation	97%		60		5	[329]

## Functionalized Metal Oxide Nanomaterials as Potential Sorbents for Contaminant Removal

Metal oxides have remained in the spotlight for researchers working in areas such as chemistry, physics, and polymer sciences. Metal oxides can inhibit the nanosized structural geometry exhibiting metallic, semiconductor, or insulating characteristics. These metal-based nanoparticles are exceptionally mobile in permeable media because of their small size and high reactivity due to the very high surface-to-volume ratio [330–332]. The high surface area to mass significantly enhances the sorption limits of nanosorbent materials. Because of their facile synthesis, high efficiency, and simplicity of characterization, nanoparticles have been increasingly explored in recent years [333]. Metal nanoparticles have magnificent electrical and optical properties, reactivity, and solid mechanical quality, and thus offer an incredible opportunity to create NM-based sensors and devices for observing environmental contamination in air, water, and soil [334]. These include iron [335], aluminum [336], titanium [337], and zinc [338].

### Single Metal Oxide Nanomaterials

The most frequently utilized form of metal oxide nanoparticles comprises a single kind of metal in its oxide form. Some of the single metal oxides that can serve as potent agents for environmental remediation purposes are discussed in detail below.

#### Iron Oxide Nanoparticles

Among the various metal oxides, iron oxide nanoparticles are widely used in pollutant remediation process [339]. Iron oxide-based NMs are further divided into three forms: magnetite (Fe_3_O_4_), maghemite (Fe_2_O_3_), and hematite (Fe_2_O_3_) nanoparticles. These nanoparticles possess unique features such as high saturation magnetization, vast surface area, and a large number of active sites for the sorption of metals. Also, the magnetic properties facilitate the isolation of the magnetic nanoparticles from an aqueous medium [340, 341].

#### Magnetite (Fe_3_O_4_) Nanoparticles

**Fig. 10:**
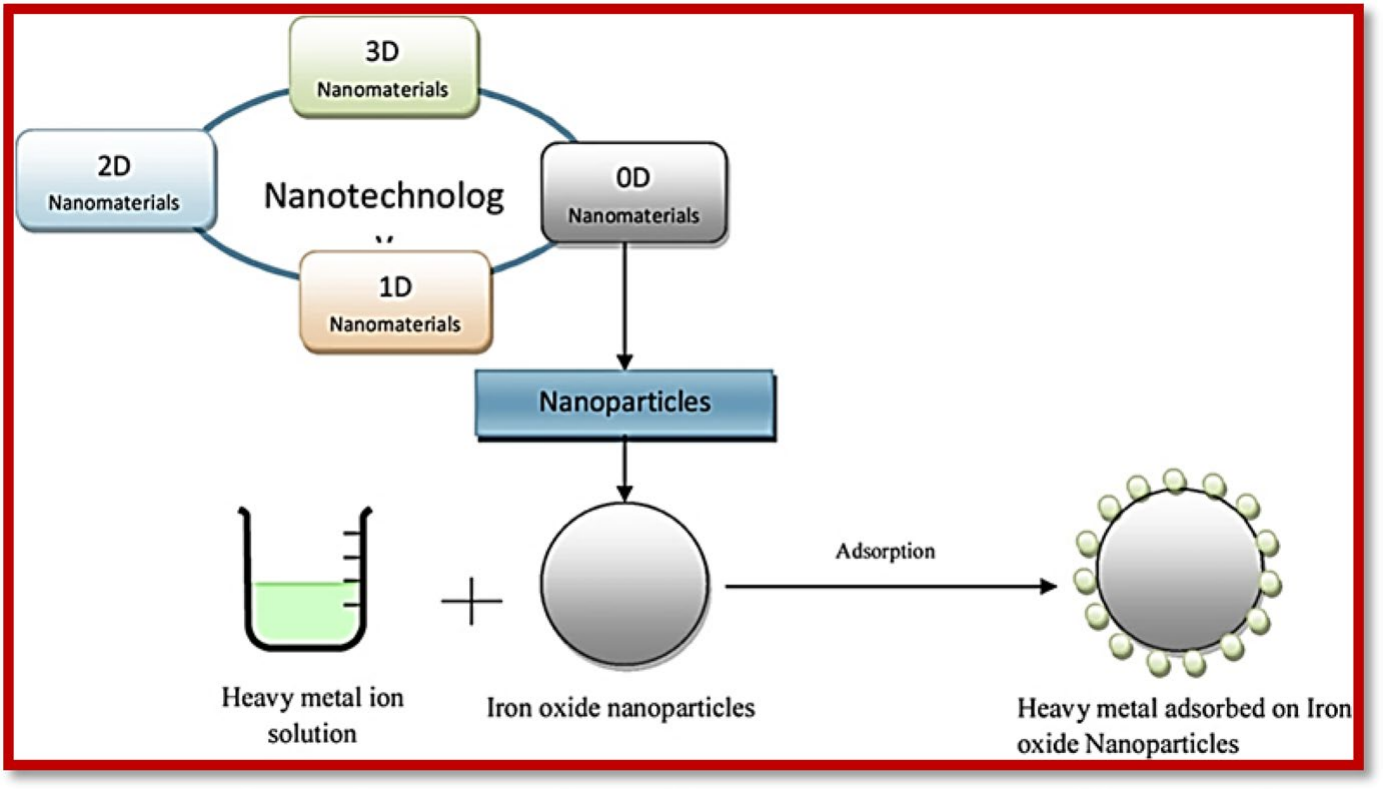
Illustration of 2D magnetic iron oxide nanoparticles for heavy metal removal. Reproduced with permission [345], Copyright 2019, Elsevier

Iron oxide/magnetite nanoparticles possess an opposite spinel structure, with oxygen having a cubic cluster, with half of Fe(III) cations having tetrahedral sites and others having octahedral sites [342]. The magnetite is ferrimagnetic in nature and possesses superexchange oxygen-mediated coupling, thus allowing the iron particles to have inverse magnetic moment directions. In magnetite, the Fe(III) quantity is similar in all cross-sectional sites; hence their magnetic moment cancels each other. As a result, the net polarization is attributed to Fe(II) cations [343]. The features of these magnetic NMs change drastically from mass to nanometer size. As the size decreases, the attractive material changes from a multi-domain structure to a solitary area structure, with novel magnetic properties. These magnetic nanoparticles show amphoteric surface action, simple scattering capacity, and a high surface-to-volume ratio, affording high metal ion sorption capacity [344]. Magnetite nanoparticles are further stabilized by various organic/inorganic supports and then used in the sorption and photocatalytic degradation of environmental contaminants [345] (Fig. 10).

Song et al. [346] exploited an iron oxide-activated persulfate system for the simultaneous removal of Cr(VI) and triclosan (TCS). The operation was based on the utilization the sulfate radicals (SO_4_^**·**−^) as efficient substances for the degradation of triclosan along with the removal of Cr(VI). At optimized conditions, the removal efficiency obtained for Cr(VI) removal was 99.5%, while 87.5% degradation of TCS was achieved. The possible mechanism was that Fe(II) from iron oxide activation of persulfate produces SO_4_^**·**−^ radicals that react with water, producing OH^**·**^. This hydroxyl radical will cause the degradation of triclosan. At the same time, Cr(VI) is sorbed on the surface of iron oxide, causing the reduction reaction of both Cr(VI) and Fe(II), resulting in the oxidation of iron to Fe(III) and reduction of chromium to Cr(III) [347]. The final forms of both Fe(III) and Cr(III) will co-precipitate on the iron oxide surface, forming Cr–Fe (oxy) hydroxide. This indicates the successful removal of both contaminants.

D’Cruz et al. [348] prepared iron oxide nanoparticles coated with activated carbon (AC-Fe_3_O_4_composite) as a potential sorbent for the removal of an antipsychotic drug, promazine, from wastewater. The results revealed complete removal of promazine, wit sorption capacity of 101.1 mg/g, within a period of 6 min. This fastest and highest removal efficiency (99.9%) was attributed to the electrostatic interactive forces created between the sorbent and the sorbate. This mechanism was determined by optimizing the pH and creating a range of isoelectric points for the nanocomposite and promazine. The studies revealed that below pH 9.3, the sorbent is positively charged, hence promoting attractive forces for the negatively charged promazine particles, exhibiting better sorption ability.

Magnaccaet al. [349] examined the efficiency of Fe_3_O_4_ nanoparticles (NPs) coated with soluble bio-based products (SBO) as prospective sorbents for the removal of pollutants in wastewater. The biosorbent was prepared following the co-precipitation method [350], and the prepared particles had a diameter of 10 nm. CV dye was used as a target to check the sorption efficiency of the prepared SBO-coated nanoparticles. The results showed greater efficiency, as the useful properties of both nanoparticles and a bio-based product were incorporated. NPs with different amounts of SBO were tested, and NP/0.5 demonstrated the highest sorption capacity of 85%. This was attributed to the fact that higher negative charges on the surface of NP/0.5 exhibited greater attraction towards the cationic CV dye, leading to better sorption at neutral pH.

Giri et al. [351] prepared magnetic nanoparticles from waste iron ore tailings by co-precipitation of its aqueous acidic solution along with ferrous iron under an inert atmosphere. The prepared magnetic nanoparticles (MNPs) were utilized for the sorption of MB and Congo red (CR) dyes to evaluate the removal efficiency of the prepared MNPs. The MNPs showed greater sorption capacity of 70.4 mg/g and 172.4 mg/g for MB and CR, and hence proved to be an excellent sorbent. The fast rate of sorption was ascribed to the absence of any internal diffusion, with the sorption occurring only on the surface of the MNPs. The sorption ability had a considerable influence on the pH of the medium, which followed different trends for the two dyes. The increase in pH results in a negative charge on the surface of the NPs, leading to enhanced sorption of the cationic dye (MB) and decreased sorption of anionic dye (CR). In contrast, increased sorption was observed at low pH for anionic dye due to the development of a positive charge at lower pH.

#### Maghemite (γ-Fe_2_O_3_) Nanoparticles

There are many reports of organic polymer-supported maghemite nanoparticles. Nanosized iron oxide particles are widely used in different industrial processes including the manufacture of semiconductors, recording materials, catalysts, and gas sensor materials [252]. Afkhami and Moosavi [352] prepared maghemite nanoparticles by a co-precipitation method and assessed the efficiency of the prepared nanoparticles for the removal of CR dye. The highest sorption capacity was 208.33 mg/g (pH ~ 5.9). The mechanism of greater sorption efficiency of the dye was explained based on the pH of zero-point charge, pH_zpc_, where below pH_zpc_, the surface of the sorbent is positively charged, making it available for the sorption of anionic dye. Additionally, CR and metal oxides develop a coordination effect, leading to sorption.

Behera et al. [353] studied the removal of hexavalent chromium based on sodium dodecyl sulfate (SDS)-modified maghemite nanoparticles. The highest removal efficiency of 95.8% was obtained for chromium at a pH of 2.6. The study of the sorption of chromium at lower pH showed that, at lower pH, chromium exists in various oxyanion forms including H_2_CrO_4_, HCrO_4_^−^, CrO_4_^2−^, and Cr_2_O_7_^2−^. At lower pH values, the H^+^ could be sorbed to SO_4_^−^ ions of the SDS and form complexes with Cr_2_O_7_^2−^ and HCrO_4_^−^ through electrostatic interactions, indicating the removal of hexavalent chromium ions. Minisy et al. [354] prepared poly(*p*-phenylenediamine)/maghemite (PPDA/γ-Fe_2_O_3_) composites by oxidative polymerization. The prepared sorbent exhibited excellent sorption capacity towards Reactive Black 5, an anionic dye. The highest sorption capacity obtained was 223 mg/g at the optimized working conditions. The positively charged PPDA-doped maghemite tends to attract the anionic Reactive Black 5 dye, promoting efficient sorption. The sorption mechanism tends to follow the electrostatic interaction along with the π-π stacking of the aromatic rings.

#### Hematite (α-Fe_2_O_3_) Nanoparticles

Hematite nanoparticles can also be used as sorbents for the removal of various environmental pollutants because of their properties such as stability at ambient conditions and environmentally friendly n-type functional material and semiconductor [355]. Kefeniet al. [356] studied the synthesis of hematite nanoparticles and utilized them as sorbents for the removal of various metal ions from acid mine drainage (AMD) confirming the efficiency of these nanoparticles for wastewater remediation. The highest efficiency of up to 80% was achieved for the removal of various metal ions. The possible pathway for the removal of metal ions in the sorption and the formation of various metal oxides on the surface of the hematite resulted in its removal. Saadet al. [357] reported a novel hematite@chitosan core/organic shell nanocomposite (HCS) to remove Pb(II), Cu(II), and Cd(II) ions from industrial wastewater. Both hematite nanoparticles and chitosan have unique sorption properties. Hematite nanoparticles have a high surface area and high saturation magnetization, while chitosan is a naturally occurring polysaccharide and has excellent properties for the sorption of metal ions by ion exchange as well as by coordination linkage mainly due to the presence of the –NH_2_ group in the chitosan matrix [358]. The highest sorption capacity obtained for Pb(II), Cu(II), and Cd(II) was 476.1 mg/g, 117.6 mg/g, and 135.1 mg/g, respectively.

#### Titanium dioxide (TiO_2_) Nanoparticles

Titanium dioxide (TiO_2_) and zinc oxide (ZnO) are widely used for photocatalytic activity. Titanium dioxide is a photocatalyst that has been used in solar cells, paints, and coatings. TiO_2_ in the anatase phase has been of particular interest due to its high oxidization power for organic contaminants, chemical stability, and low cost [359]. It is commercially used as a photocatalyst [360]. Supporting elements such as sand, glass, or zeolite enhance the separation efficiency of nanocrystalline TiO_2_. Magnetic separation provides a very convenient approach for removing and recycling magnetic particles such as magnetite, ferrite, and barium ferrite by applying external magnetic fields. The incorporation of magnetic components into TiO_2_ nanoparticle-based catalysts may therefore enhance the separation and recovery of nanosized TiO_2_ [361]. Photocatalysis appears to be a very efficient pretreatment process for wastewater streams containing organic matter.

**Fig. 11:**
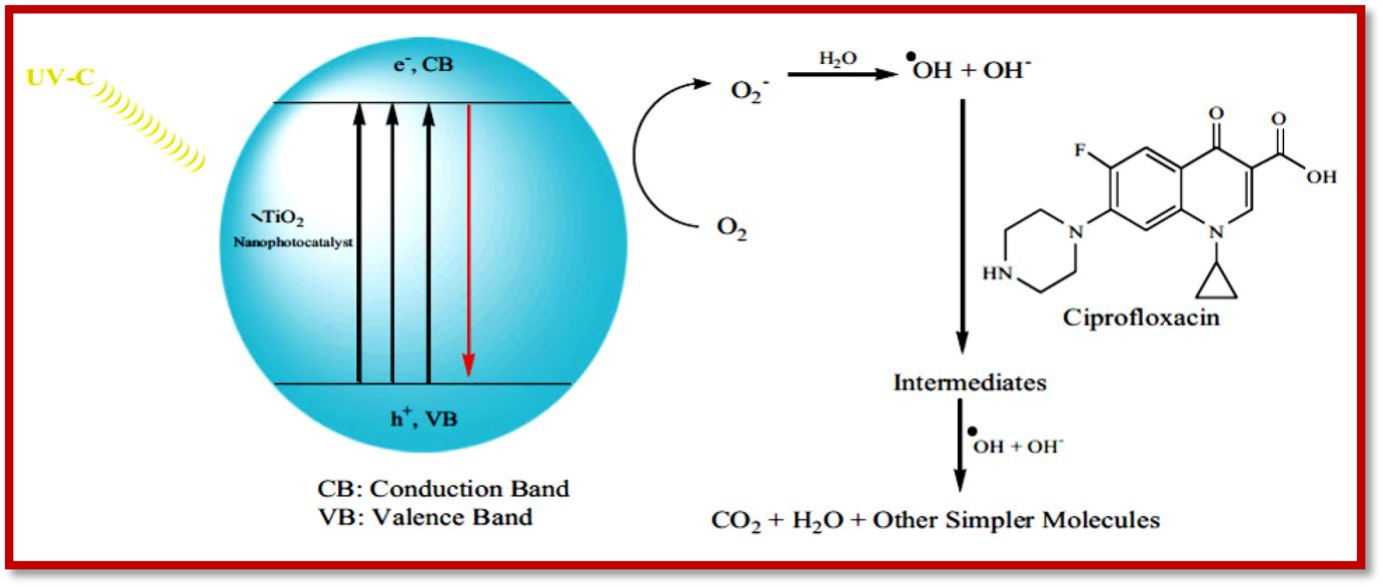
A schematic explanation of UV irradiation for ciprofloxacin degradation on TiO_2_ immobilized on the surface of a glass plate. Reproduced with permission [362] Copyright 2020, Informa UK Limited

Malakootian et al. [362] analyzed the photocatalytic degradation efficiency of TiO_2_ immobilized on the surface of a glass plate for ciprofloxacin. The benefit of using glass as a supporting agent for TiO_2_ is to enhance the life span as well as the reusability of the catalyst. The degradation of ciprofloxacin begins with the production of e^−^/h^+^ pairs when an illumination source is provided (Fig. 11). These pairs of e^−^/h^+^ cause the generation of hydroxyl radicals or other radicals by reaction with H_2_O. These produced radicals act as oxidizing agents for the degradation of ciprofloxacin. Another possibility for the degradation of ciprofloxacin is the direct reaction of the produced holes with the ciprofloxacin. Additionally, the e^−^ tends to reduce ciprofloxacin or produce superoxide radicals (O_2_^−**·**^) that can mineralize the contaminants [363].

### Binary Metal Oxide Nanomaterials

One process that has drawn attention is the consumption of more than one metal in a combinational form to utilize the useful properties associated with each one of them promoting better results. Two or more metals incorporated as one compound are widely used for wastewater decontamination purposes due to the increased surface area, parallel redox reactions/ion-exchange reactions, and reduced agglomeration probability, eventually enhancing the sorption rate as well as the degradation rate as sorbents or photocatalysts, respectively [364]. Binary metal oxides generally represented as M_1_/M_2_O are a continuation of this approach.

Mohanta et al. [365] studied the preparation of iron-zirconium binary oxide (IZO) as an efficient sorbent through the co-precipitation method. The prepared material had a surface area of 200.307 m^2^/g, which is sufficient for maximum sorption. The prepared sorbent was utilized for the removal of CR, an anionic dye, and showed greater efficiency, with sorption capacity of 171 mg/g. The removal of the dye was attributed to the electrostatic interactions and hydrogen bonding between the sorbate and sorbent molecules. Once the pH of the solution is decreased, the surface of the sorbent becomes positively charged and the H^+^ concentration in the solution is quite high. This enables the convenient removal of the anionic dye, CR in this case.

Du et al. [366] performed phosphate ion removal by preparing a bimetallic nanocomposite, incorporating a binary metal oxide (La-Zr) into the porous structure of a polymeric anion exchanger (D201). The obtained La-Zr-D201 offers specific sorption of the phosphate ions through a ligand exchange strategy. The highest sorption capacity obtained by the prepared ion-exchanger was noted as 61.31 mg/g, while phosphate treatment capacity of 1350 BV was also obtained.

### Ternary Metal Oxide Nanomaterials

Ternary metal oxide nanoparticles comprising three metals are also trending for the removal of the contaminants. Ghasemipour et al. [367] prepared ternary nanocomposites for the photocatalytic degradation of aniline by doping ZnO onto MoS_2_, followed by grafting on reduced graphene oxide and carbon nanotubes. The coupling of the transition metal sulfide inhibits the recombination of the charges, hence enhancing the photocatalytic activity. The incorporation of rGO and CNTs further enhances the catalytic activity. The results showed that at the optimized conditions, the photocatalytic activity of rGO10%/ZnO20%/MoS_2_ and CNT10%/ZnO20%/MoS_2_ was 84% and 76%, respectively. The difference in the photocatalytic efficiency between the catalysts was attributed to the sorption efficiency of the prepared photocatalyst, which is greater for the rGO-modified photocatalyst than the modified CNTs due to their larger *S*_BET_ values.

Eniola et al. [368] also prepared binary and ternary metal hydroxides and utilized them for the removal of an antibiotic, oxytetracycline. The binary and ternary hydroxides were made using copper, aluminum, and manganese metals as binary CuAl-hydroxide, MnAl-hydroxide, and ternary CuMnAl-hydroxide. The sorption capacity of the prepared sorbents was in the decreasing order of CuMnAl-hydroxide > CuAl-hydroxide > MnAl-hydroxide. The mechanism for the sorption of the antibiotic drug was attributed to the electrostatic interaction, hydrogen bonding, and anion exchange. FTIR studies were performed to confirm the sorption of OTC onto the surface of metal hydroxides and predicting the mechanism of sorption. The results showed a decrease in the band intensities and a change in position of the bands of the metal hydroxide after sorption as compared with before sorption material, predicting the electrostatic interaction between the OTC and MH. The OH band sharpness was considerably reduced indicating the presence of hydrogen bonding after sorption, while peaks of exchangeable ions such as SO_4_^2−^ disappeared, indicating the anion exchange between the sorbent and the sorbate, confirming the sorption efficiency of the prepared materials. The highest sorption capacity possessed by the ternary hydroxide was found to be 250.07 mg/g.

### Other Metal Oxide Nanoparticles

Apart from iron and titanium oxides, several other metal oxides have been exploited to date for the removal of various contaminants [369, 370]. These metal oxides exhibited greater contaminant removal properties based on the small size and higher surface-to-volume ratios. These metal oxides include ZnO [371], CuO [372], Al_2_O_3_ [373], CeO_2_ [374], SiO_2_ [375]_,_PbO [376], and SnO_2_ [377], to name a few.

Debnath and Mondal [378] followed a green approach for the synthesis of zinc oxide nanoparticles using leaf extracts of *Hibiscus rosa-sinensis* with the aim of reducing the cost and complexity associated with the commercial methods of nanoparticles preparation. ZnO has been frequently utilized as a potent photocatalyst based on its wide bandgap of 3.37 eV; hence, it could be used in UV [379]. At present, its sorptive properties have also been explored against various contaminants. Here, the prepared ZnO nanoparticles were used for the removal of CR dye. Removal efficiency of 95.5% was exhibited by the prepared ZnO nanoparticles for the removal of CR dye in 20 min contact time at a pH of 4. The possible mechanism of the sorption was explained based on electrostatic interaction forces between the ZnO and the azo group of the dye. The amine group of dye molecules may exhibit some attraction towards the ZnO nanoparticles, leading to efficient removal of the dye.

Rafique et al. [380] investigated the green synthesis of copper oxide nanoparticles by utilizing leaf extract of *Citrofortunella microcarpa* (Calamondin) for the efficient removal of RB. CuO exhibits a bandgap of 1.35 and 3.5 eV and has proved to be efficient semiconductors suitable for performing the photocatalytic degradation of wastewater contaminants [381]. The prepared CuO nanoparticles exhibited excellent efficiency, with photocatalytic degradation of RB dye up to 98% at the optimized conditions. The mechanism for the removal of dye through photocatalytic degradation is shown in Eqs. (1–8):

1 $$\mathrm{CuO}+h\vartheta \to {\mathrm{e}}^{-}\left(\mathrm{CBCuO}\right)+ {h}^{+}\left(\mathrm{VBCuO}\right)$$

2 $$2\mathrm{CuO}+{\mathrm{H}}_{2}\mathrm{O}+ {\mathrm{e}}^{-}\to 2\mathrm{Cu}+2{\mathrm{OH}}^{-}$$

3 $$2\mathrm{CuO}+2{\mathrm{OH}}^{-} \to 2\mathrm{CuO}+ {\mathrm{H}}_{2}\mathrm{O}+2{\mathrm{e}}^{-}$$

4 $${\mathrm{e}}^{-}\left(\mathrm{CBCuO}\right)+ {h}^{+}\left(\mathrm{VBCuO}\right)\to \mathrm{recombination}$$

5 $${\mathrm{e}}^{-}+ {\mathrm{O}}_{2} \to {\mathrm{O}}_{2}^{-} + {\mathrm{H}}_{2}\mathrm{O }\to {\mathrm{OH}}_{2\bullet } +{\mathrm{OH}}^{- }$$

6 $${h}^{+} + {\mathrm{OH}}^{-} \to {\mathrm{OH}}^{\cdot }$$

7 $${\mathrm{OH}}^{\cdot }+\mathrm{ RhB \,dye }\to \mathrm{degradation\, products}$$

8 $${\mathrm{h}}^{+} +\mathrm{dye}\to \mathrm{dye oxidation }\to \mathrm{degradation}$$

The radicals produced in the above reactions are mainly responsible for the degradation of the dye.

Zhang et al. [382] studied the preparation of γ-Al_2_O_3_ nanoparticles through a hydrothermal process for the successful removal of anionic CR dye. The highest sorption capacity exhibited by the prepared sorbent for the removal of the CR dye was 465.82 mg/g. A comparison was made to evaluate the sorption capacity of the γ-Al_2_O_3_ nanoparticles by evaluating the sorption of other cationic dyes such as MB and malachite green. The results indicated that the sorbent demonstrated poor sorption capacity for the cationic dyes as compared to the CR. This was explained based on the presence of sulfonate groups on the surface of the CR dye in the form of SO_3_^−^, which gave rise to electrostatic interactions with the surface of the sorbent dye, leading to efficient removal. Another factor involved in the removal of the dye is the presence of an amino group that promotes the hydrogen bonding with the hydroxyl group, leading to their strong interaction. Additionally, the azo bonds present on the surface of the CR dye leads to the development of a hydrogen bond with the hydroxyl group. Hence, the sorption of dye is attributed to both the electrostatic and hydrogen bonding forces between the dye and the sorbent particles.

Liu et al. [383] also synthesized cerium oxide nanoparticles laminated with lignin for the efficient removal of phosphate. The prepared L-NH_2_@Ce exhibited a surface area of 89.8 m^2^/g and a pore volume of 0.23 cm^3^/g. The sorption capacity exhibited by the prepared sorbent was 27.86 mg/g. The exceptionally high sorption capacity was attained at mildly acidic conditions with pH 5. This might be because, in alkaline conditions, there is competition between OH^−^ and PO_4_^3−^ ions for attaching to the surface of the sorbent, leading to reduced sorption of phosphate. Li et al. [384] fabricated an anion exchange resin D201, with nanosized hydrous zirconium oxide (HZrO) for successful removal of vanadium(V). Favorable sorption capacity of 118.1 mg/g was achieved for the removal of V(V), confirming the excellent capability of the prepared HZrO@D201HZrO@D201 sorbent. The removal of the contaminant was attributed to the nonspecific ion exchange and electrostatic interactions between the resin and the V(V). The resin incorporated in the prepared sorbent is a macroporous anionic resin that can attract negatively charged ions, while the zirconium lamination exhibits a positive charge, which also promotes the negative charge attraction through electrostatic interaction at high pH, thus promoting the sorption of V(V) (Table 4).

**Table 4:** Metal oxide nanomaterial in wastewater remediation

Nanomaterial	Contaminant	Mechanism	Sorption/catalytic capacity	Conditions studied			Cycles	References
				pH	Time	Temp °C		
Magnetic nanoparticles composite with silestone and biochar (MNPs/EDB/SS)	Cd	Sorption	117.38 mg/g	8	120	25		[385]
Magnetic nanoparticles activated with biochar (QBC/MNPs)	Cr(VI)	Sorption	77.35 mg/g	4	180		5	[386]
Magnetic nanoparticles	Cd(II)	Sorption	42.3 mg/g	6				[387]
	Pb(II)		42.5 mg/g					
	Zn(II)		42.9 mg/g					
Magnetic nanoparticles coated with surfactants (MNPs-CPC)	Sb(V)	Sorption	113.63 mg/g	4.3		25		[388]
Magnetite-functionalized boron nitride nanosheets (BNNS-Fe_3_O_4_)	As(III)	Sorption	30.3 mg/g	8	500		5	[389]
Iron oxide incorporated with mesoporous biochar	Ofloxacin	Sorption	19.74 mg/g	6	300	25	5	[390]
Monolayers of *N*-(2-aminoethyl)-3-aminopropyltrimethoxysilane onto magnetic nanoparticles (MSM)	Cr(VI)	Sorption	8 mg/g	4	120		5	[391]
Magnetite								
M1	Pb & Cd	Sorption	408.14 and 228.05 mg/g	6	1440	56	6	[392]
M2			331.40 and 170.86 mg/g					
M3			178.47 and 83.49 mg/g					
Magnesium-doped magnetic nanoparticles	As(V)	Sorption	33.71 mg/g	8–11		25	5	[393]
Magnetic nanoparticles embedded Gum-ghatti-graft-poly(4-acryloylmorpholine) hydrogel (Ggh-*g*-PAcM/Fe_3_O_4_)	Methylene blue	Sorption	116.8 mg/g	7	45	30	1	[394]
	Rhodamine B		137.8 mg/g					
	Cu(II)		249.9 mg/g					
	H(II)		235.1 mg/g					
Three-dimensional (3D) magnetic bacterial cellulose nanofiber/graphene oxide polymer aerogel (MBCNF/GOPA)	Malachite green	Sorption	270 mg/g	12	25	25	8	[395]
Chitosan coated magnetic nanoparticles	Copper	Sorption	1.03 mg/g	6	90		5	[396]
	Chromium		0.20 mg/g					
	Arsenic		0.04 mg/g					
	Phenol		0.56 mg/g					
Tin magnetic nanocomposites (Sn-CCMN)	Alizarin yellow	Sorption	92%	7	120	25	4	[397]
ZnO-magnetic/ZSM-5	Disperse blue 56	Sorption	6.23 mg/g	3	15		5	[398]
NiFe_2_O_4_-COF-chitosan-terephthalaldehyde nanocomposites film (NCCT)	Tetracycline	Sorption	388.52 mg/g	8			6	[399]
	Cefotaxime		309.26 mg/g	4				
Magnetic magnetite nanoparticles	Boron	Sorption	4.57 mmol/g	5	300	45	3	[400]
Biochar-modified iron oxide nanoparticles (MBC)	Caffeine	Sorption	75.1 ± 1.8 mg/g	8	5	35	5	[401]
	Ibuprofen		39.9 ± 1.2 mg/g					
	Acetylsalicylic acid		149.9 ± 4.5 mg/g					
SnFe_2_O_4_/ZnFe_2_O_4_ Heterojunctions	Tetracycline	Photocatalytic degradation	93.2%		120		4	[402]
g-C_3_N_4_/NiO/ZnO/Fe_3_O_4_ nanohybrid	Esomeprazole	Photocatalytic degradation	95%	6	70		5	[403]
N-doped TiO_2_/SiO_2_-based nanomagnetic photocatalyst (N-TiO_2_@SiO_2_@Fe_3_O_4_)	Paraquat (PQ; 1,1′-dimethyl-4,4′-bipyridinium dichloride)	Photocatalytic degradation	85%	6	180		8	[404]
TiO_2_-loaded magnetic MOFs composite (TiO_2_/mag-MIL-101(Cr))	Bisphenol F	Photocatalytic degradation	90%		60		5	[405]
	Acid red 1							
Magnetic Fe_3_O_4_/ZnWO_4_/CeVO_4_ nanoparticles	Methyl violet	Photocatalytic degradation	90%					[406]
	Methylene blue		70%					
TiO_2_@ZnFe_2_O_4_/Pd nanocomposite	Diclofenac	Photocatalytic degradation	86.1%	4	120		5	[407]
Carboxymethyl-β-cyclodextrin-modified Fe_3_O_4_@TiO_2 (_CMCD-Fe_3_O_4_@TiO_2_)	Polychlorinated biphenyl	Photocatalytic degradation	83%		16	25	5	[408]
ZnFe_2_O_4_@TiO_2_/Cu nanocomposite	Naproxen	Photocatalytic degradation	80.73%	4	120		5	[409]
Fe_3_O_4_ magnetic nanoparticles (MNP)	Nitrobenzene	Photocatalytic degradation	73.13%	2	120	25		[410]
Lanthanum substituted spinel ferrite (La_x_MnFe_2-x_O_4_) nanoparticles	Crystal violet dye	Photocatalytic degradation	95%		90			[411]
Fe_3_O_4_/TiO_2_/CuO nanoparticle	Methylene blue	Photocatalytic degradation	99%	7	22		4	[412]
TiO_2_ magnetic nanoparticles (T-MNPs)	Ceftazide	Photocatalytic ozonation	75.5%	11	15		6	[413]
Core/shell nanocomposite of phosphomolybdic acid immobilized on magnetic alumina (Fe_3_O_4_@Al_2_O_3_-PMo)	Cibacron brilliant yellow	Photocatalytic degradation	90%	7.2	240		5	[414]
Surface-modified hematite NPs (α-Fe_2_O_3_ NP_s_)	Pb^2+^	Sorption	111 mg/g	6.5	120	25		[415]
Hematite iron oxide NPs α-Fe_2_O_3_	Malachite green	Sorption	86.13%		45			[416]
Hematite (α-Fe_2_O_3_) nanoparticles	Cr^6+^	Sorption			30			[417]
Polyacrylonitrile (PAN) with embedded hematite (α-Fe_2_O_3_) NPs PAN/Fe_2_O_3_@Fe_2_O_3_ nanofibers	As(V)	Sorption	5 mg/g	6				[418]
	Cu(II)		19 mg/g					
	Cr(VI)		3.9 mg/g					
Disc-like hematite (α-Fe_2_O_3_) NPs	Ni^2+^	Sorption	62.5 mg/g	6				[419]
	Cd^2+^		200 mg/g					
Hematite (α-Fe_2_O_3_) NPs	Cu^2+^	Sorption	105 ± 7 mg/g	6–9.5	300			[420]
	Ni^2+^		104 ± 7 mg/g					
	Co^2+^		80 ± 6 mg/g					
	Cd^2+^		96 ± 7 mg/g					
	Pb^2+^		100 ± 0.6 mg/g					
Hematite (α-Fe_2_O_3_) NPs	Rose Bengal dye	Sorption	1810 mg/g	5		30		[421]
Nano-α-Fe_2_O_3_ NPs	Co-60	Sorption	142.86 mg/g	6.5	120	25		[422]
Hematite (α-Fe_2_O_3_) NPs	Crystal violet	Sorption	100%		30			[423]
	Methylene blue				40			
Hematite/lignosulfonate composite (HLS)	Cd(II)	Sorption	39.03–53.65 mg/g		200			[424]
Mesoporous maghemite NPs γ-Fe_2_O_3_	Methylene blue	Sorption	36 mg/g	10				[425]
Maghemite nanoparticles coated *Bacillus subtilis*	Cd^2+^	Sorption	32.6 mg/g	4	60	30		[426]
Starch-functionalized maghemite nanoparticles (γ-Fe_2_O_3_@starch)	As(III)	Sorption	8.60 mg/g	2–9				[427]
Montmorillonite-based magnetic nanoparticles (Mt@MH)	Pb(II)	Sorption	38.15 mg/g	6.5	480	25	5	[428]
	As(V)		19.10 mg/g	3.5				
Novel nanoadsorbents based on core–shell bimagnetic nanoparticles (CoFe_2_O_4_@ɣ-Fe_2_O_3_)	Cr(VI)	Sorption	15.6 mg/g	2.5	20	25	3	[429]
Chitosan coated iron oxide nanoparticles	Cr(VI)	Sorption	162.54 mg/g	3.7	21.84		7	[430]
Al-doped nano-TiO_2_	Methylene blue	Photocatalytic degradation	85%		30			[431]
TiO_2_	CN	Photocatalytic degradation	80%	10				[432]
	Cr(VI)			2.3				
TiO_2_-coated glass sheet	Methylene blue	Photocatalytic degradation	93%	11	60		20	[433]
TiO_2_ deposited on polyethylene terephthalate (PET-TiO_2_)	Carbendazim (CBZ)	Photocatalytic degradation	80%					[434]
	Caffeine (CAF)							
g-C_3_N_4_ nanosheets implanted TiO_2_ nanotube (TCNs)	Tetracycline	Photocatalytic degradation	100%		120			[435]
Zinc oxide NPs (ZnO NPs)	Methylene blue	Photocatalytic degradation	100%	7	60	25		[436]
	Rhodamine B				50			
Zinc oxide NPs (ZnO NPs)	Dibenzothiophene (DBT)	Photocatalytic degradation	97%	7	180		5	[437]
Zinc oxide NPs (ZnO NPs)	Rhodamine B	Photocatalytic degradation	98%		200			[438]
Cobalt oxide NPs (CoNPs)	Direct yellow-142	Catalytic reduction	93.37%		60			[439]
	Methyl orange		96.24%					
Magnetic cobalt oxide nanoparticles (CONP)	Malachite green	Sorption	238.10 mg/g	7	120		4	[440]
Cobalt ferrite composite nanoparticles (CoFe_2_O_4_)	Acid black 1	Photocatalytic degradation	80%		120			[441]
	Reactive red 4		61%		105			
Copper-doped ZrO_2_ NPs	Methyl orange	Photocatalytic degradation	98%		100			[442]
Copper oxide NPs (CuO NPs)	Nile blue	Photocatalytic degradation	93%		120			[443]
	Reactive yellow 160		81%					
Nickel oxide nanoparticles (NiO NPs)	Ciprofloxacin	Sorption	99.8%	3	100	25		[444]
Zinc oxide nanoparticles impregnated Pea peels (ZnONPs-IPPs)	Chlorpyrifos	Sorption	47.846 mg/g	2	60	30–50		[445]
Zinc oxide NPs	Methylene blue	Photocatalytic degradation	80%		120			[446]
Chitosan-zinc sulfide nanoparticles (CS-ZnS-NPs)	Acid brown 98	Photocatalytic degradation	92.6%	6	165	33	4	[447]
	Acid black 234		96.7%	7	100			
CoCrFeO_4_ oxide chitosan-composite beads (CoCrFeO_4_-CB)	Acid black	Photocatalytic degradation	100%		120	35	5	[448]
	Acid brown		93%					
	Congo red		85%					
SnO_2_ NPs	Methylene blue	Photocatalytic degradation	64.42%		120			[449]
Sodium iron disulfide (NaFeS_2_)	Methylene blue	Photocatalytic degradation	97%		105			[450]
	Indigo carmine		99%		45			
Binary titanium oxides (Sm_2_Ti_2_O_7_)	Rhodamine B dye	Photocatalytic degradation	94%		80			[451]

## Functionalized Metal Selenide Nanomaterials

Metal selenides belong to a class of semiconductor nanostructures called chalcogenides. Over the past decade, metal selenide nanostructures have taken nanotechnology to the next level [452]. The remarkable features of metal selenide nanoparticles include their surface-to-volume ratios, optical and field emission properties, and photocatalytic activity. Based on these properties, metal selenides are being exploited in various fields including field-effect transistors [453], light-emitting diodes (LED) [454], solar cells [455], and wastewater remediation [456]. The commonly utilized metal selenides include ZnSe [457], CdSe [458], SnSe [459], PbSe [460], FeSe_2_ [461], CuSe [462], and CdTe [463].

Ghaedi et al. [464] explored the preparation of cadmium selenide nanoparticles loaded on activated carbon (CdSe-NP-AC), utilized for the successful removal of muroxide (MO) from aqueous solution. Sorption capacity of 333 mg/g was achieved at the optimized experimental conditions. Sharifpour et al. [465] studied the preparation of starch-capped zinc selenide nanoparticles loaded on an activated carbon (ST-Zn-Se-NPs-AC) composite, which was used for the removal of basic fuchsin (BF) dye. The prepared composite demonstrated high removal efficiency, with sorption capacity of 222.72 mg/g at the optimized operating conditions.

### Binary Metal Selenide Nanomaterials

The binary metal selenides incorporate two metals in the form of selenides, providing a composite suitable for water remediation applications. Ali et al. [466] studied the photocatalytic performance of prepared chitosan-bismuth cobalt selenide hybrid microspheres for the removal of CR dye. The prepared selenide tri-composite had a narrow bandgap of 2.48 eV, while the average size of the microspheres was found to be 734 µm.

**Fig. 12:**
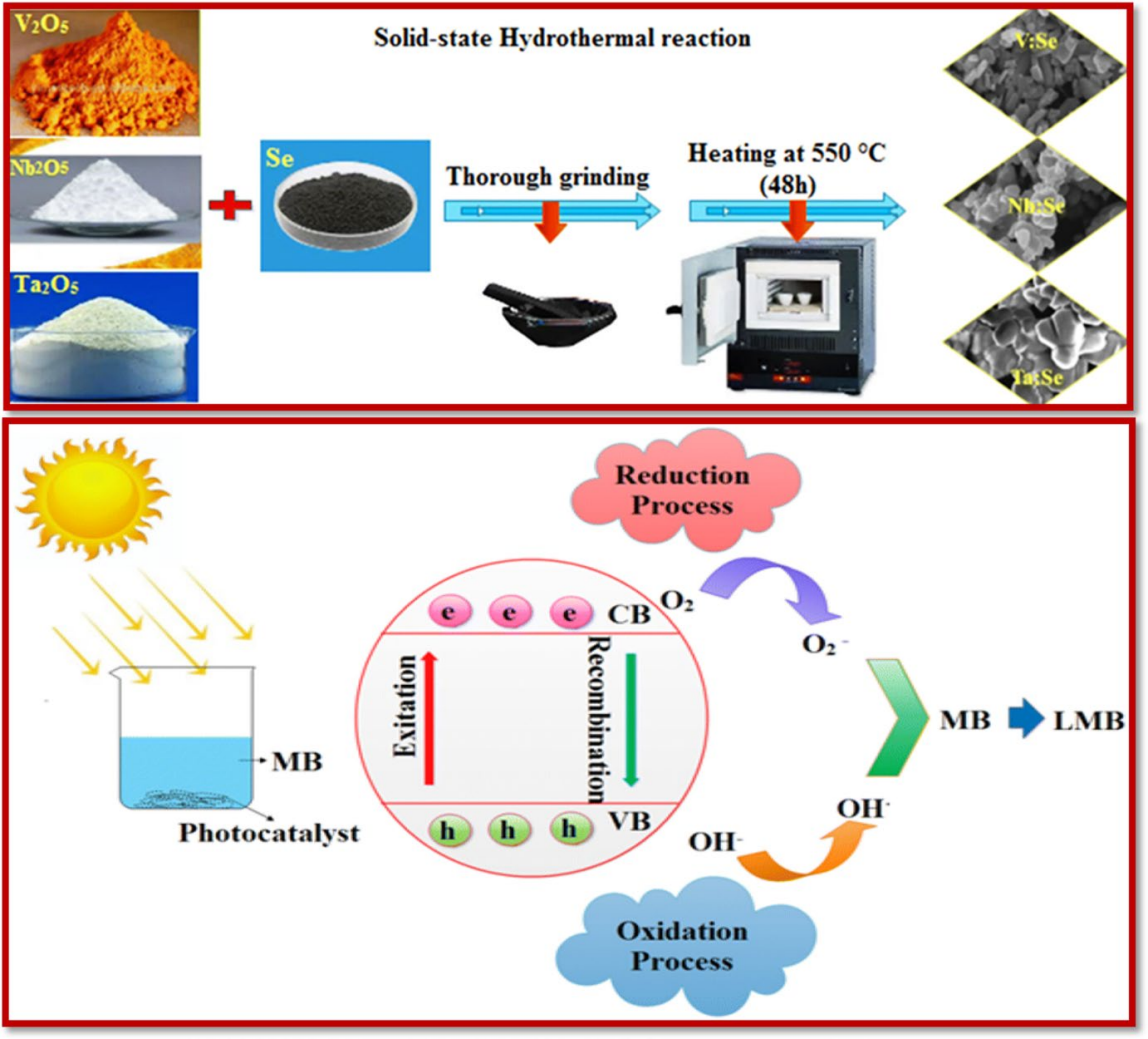
Schematic illustration of the metal selenide and their photocatalytic activity. Reproduced with permission [467], Copyright 2020, Springer Nature

The prepared BCSN-CM photocatalyst showed an excellent removal percentage of 85% for the CR dye at the optimized conditions and was successfully reused for up to five successive cycles. The idea of utilizing chitosan as a capping agent for the metal selenides nanoparticles was to avoid the leaching of the catalyst. The mechanism of the degradation of the CR dye was based on the redox reactions taking place on the surface of the catalyst, and most of the degradation was associated with the production of ^**·**^OH and O_2_^**·**−^ radicals according to the typical photocatalytic mechanism. Altaf et al. [467] explored binary transition metal selenide (V_3_Se_4_), (Nb_2_Se_3_, Nb_2_Se_9_), and (TaSe_3_, Ta_2_Se_3_) preparation through the hydrothermal method. The prepared catalyst was used for the photocatalytic degradation of MB. The prepared photocatalyst had wider bandgap energy of 3.87, 3.82, and 3.95 eV, respectively, for each semiconductor photocatalyst. Degradation efficiency of up to 90% was obtained. Figure 12 explains both the synthesis and photocatalytic activity of binary transition metal selenides (V_3_Se_4_), (Nb_2_Se_3_, Nb_2_Se_9_), and (TaSe_3_, Ta_2_Se_3_).

### Ternary Metal Selenides

Attention has recently been focused on the incorporation of multiple metals for the design of ternary metal selenides. The idea of designing ternary metal selenides provided the opportunity to incorporate different metals for achieving better and refined results. Nisar et al. [468] prepared ternary metal selenide/chitosan microspheres and utilized them for evacuating Alizarin Red S dye. The prepared TMS-CMs were morphologically found to have an average diameter of 33 nm. The prepared photocatalyst had a bandgap of 1.8 eV and presented excellent photocatalytic degradation with efficiency of up to 95.4%. The reaction followed a first-order kinetics model (Table 5).

**Table 5:** Metal selenide-based nanomaterial in wastewater remediation

Nanomaterial	Contaminant	Mechanism	Sorption/catalytic capacity	Conditions studied			Cycles	References
				pH	Time	Temp °C		
Iron-bismuth selenide chitosan microspheres	Crystal violet	Photocatalytic degradation	98.95%	8	150		4	[469]
Selenium-functionalized metal–organic framework MIL-101 Se/MIL-101	Mercury	Sorption	148.19 mg/g		90			[470]
Copper selenide-functionalized polyurethane sponge Cu_2_Se/PUS	Mercury	Sorption	25.90 mg/g					[471]
CdSe (cadmium selenide)-decorated graphene composites coupled with TiO_2_ (titanium oxide)	Methyl orange	Photocatalytic degradation	85%		180		4	Ghosh et al. [472]
	Rhodamine B							
Selenized magnetic nanoparticles (Fe_3_O_4−x_Se_y_)	Mercury	Sorption	98.1%		120	100	10	[473]
Nanosized copper selenide	Mercury	Sorption	210.8 mg/g		90	50		[474]
CuSe/ZIF-8	Mercury	Sorption	309.8 mg/g		240	50		[475]
Selenide-coated copper (Cu_2_Se-Cu)	Mercury	Sorption	100%		120	60		[476]
Molybdenum selenide nanosheets	Mercury	Sorption	1000 mg/g					[477]
Amorphous molybdenum selenide intercalating magnetite [MoSe_*x*_(inter)Fe_3_O_4_]	Mercury	Sorption	100%		240		10	[478]
Marcasite-type metal selenides (MSe_2_)	Mercury	Sorption						[479]
Zinc selenide sulphide composite ZnSe_0.7_S_0.3_	Mercury	Sorption	99%		120	150		[480]
Selenide-functionalized mineral sulfide *x*Se-FeS	Mercury	Sorption	87%		120	90	4	[481]
C fibers@MoSe_2_ nanoplate core–shell composite	Methylene blue	Photocatalytic degradation			80			[482]
	Rhodamine B							
	*p*-Chlorophenol (4-CP)							
	Potassium dichromate							
Amorphous MoSe_*x*_	Rhodamine B	Photocatalytic degradation	96.7%		120			[483]
	Methylene blue		98.9%					
MoSe_2_/TiO_2_ nanofibers	Rhodamine B	Photocatalytic degradation					3	[484]
	Tetracycline hydrochloride							
	K_2_Cr_2_O_7_							
Copper selenide	2-Chlorophenol	Photocatalytic degradation	100%					[485]
CuSe nanoparticles	Methylene blue	Photocatalytic degradation	76%		720	90		[486]
	Rhodamine B		87%					

## Functionalized Metal Sulfides Nanomaterials

Another diverse class of NMs consists of metal sulfides, which exist in nature in the form of minerals. They are cheap, abundant, and easily available entities, and hence are widely used by researchers in the various fields. Functionalization of the metal sulfides further enhances their ability, making them suitable for use in the field of environmental remediation [487].

### Single Metal Sulfide Nanoparticles

The majority of metal sulfides have been used to date based on their useful properties. Some of the commonly utilized ones include FeS_2_ [488], CoS [489], CuS [490], Ag_2_S [491], ZnS [492], and Bi_2_S_3_ [493].Wang et al. [494] prepared tin sulfide using Sn^2+^ as a tin source, an oxidizer (H_2_O_2_), a sulfur source (l-cysteine) to form SnS_2_ nanosheets with a 10 nm thickness. The prepared nanosheets were used for the sorptive removal of RB dye and showed excellent efficiency with a sorption capacity of 200 mg/g. The greater sorption rate was achieved for the removal of RB dye through electrostatic interactions formed between the cationic dye and the negatively charged surface of the sorbent. The decreased sorption with time was attributed to the covering of the active sites by the dye molecules resulting in repulsion instead of attraction leading to decreased sorption.

**Fig. 13:**
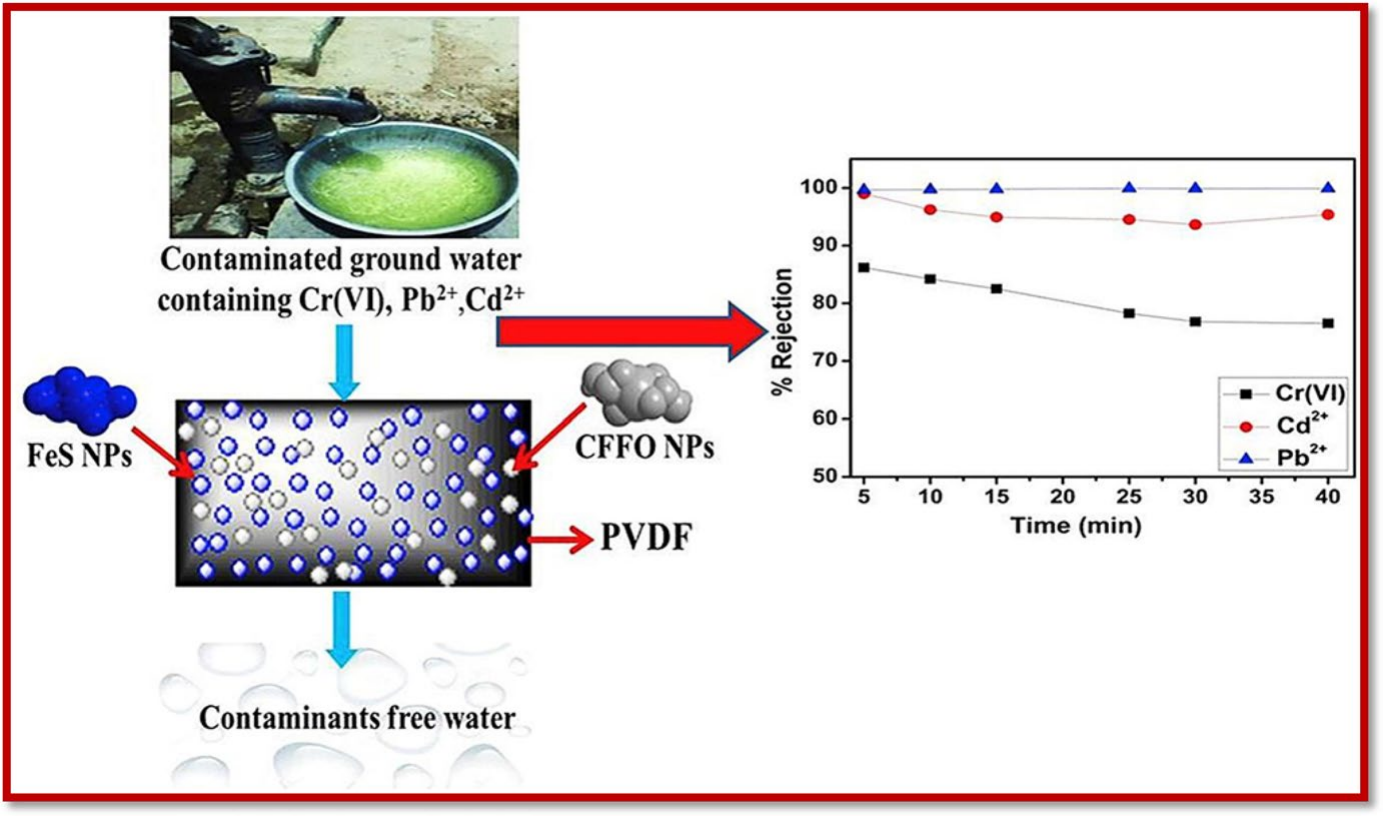
Schematic explanation of the removal of toxic heavy metal from wastewater through PVDF-based nanocomposite membranes. Reproduced with permission [495], Copyright 2020, Elsevier

Mishra et al. [495] developed ferrous sulfide (FeS) and carboxyl-functionalized ferroferric oxide (CFFO) nanoparticles, which were introduced into the polyvinylidene fluoride (PVDF) matrix (individually/mixed in an optimal ratio) following the phase inversion technique. The morphological evidence showed that both FeS and CFFO nanoparticles had a surface area of 7.22 and 89.2m^2^/g respectively, while the pore volume was recorded to be 0.382 and 0.031cm^3^/g. The prepared FeS/CFFO/PVDF membrane was then utilized for the removal of heavy metal ions particularly Pb, Cd, Cr, and As the obtained removal efficiency for the said metal ions was 88% for Cr(VI), 99% for Cd^2+^, 99% for Pb^2+^ and 95% for As(Fig. 13).

Sun et al. [496] prepared nanoscale FeS-Fe_3_O_4_ nanocomposites using chitosan as a stabilizer (CTO-MFeS). The as-synthesized nanocomposite had a size of 20 nm, while the specific surface area was recorded to be 21.3m^2^/g. The CTO-MFeS were used for the removal of Hg^2+^ ions based on the sorption as well as the precipitation phenomena. The highest sorption capacity obtained for the removal of mercury ions was noted to be 72.34 ± 3.18 mg/g.

### Binary Metal Sulfide Nanomaterials

Binary metal sulfides are also trending based on the idea of fusing the properties of both the metals being used, enhancing the activity of the tailored material. Yu et al. [497] prepared strontium titanate/binary metal sulfide (SrTiO_3_/SnCoS_4_) heterostructure through a two-step hydrothermal method. The efficiency of the prepared nanocomposite was checked by performing the photocatalytic degradation of methyl orange dye under visible light. The results showed 95% degradation confirming the potential of the photocatalyst for environmental remediation purposes.

Kalpana and Selvaraj [498] developed novel ZnS/SnS/A-FA nanorods by providing pristine reactants(fly ash as supporting material, zinc nitrate hexahydrate, stannous chloride dihydrate, and sodium sulfide) at ambient temperature. According to the Brunauer–Emmett–Teller (BET) analysis, the ZnS/SnS/A-FA nanorods have a specific surface area of 93.73 m^2^/g. The prepared ZnS/SnS/A-FA nanorods were used for the photocatalytic degradation of CR dye. The results obtained confirmed the removal efficiency of about 90% by the prepared nanorods (Table 6).

**Table 6:** Metal sulfide-based nanomaterial in wastewater remediation

Nanomaterial	Contaminant	Mechanism	Sorption/catalytic capacity	Conditions studied			Cycles	References
				pH	Time	Temp °C		
CuS/K_2_S_2_O_8_	Orange II	Photocatalytic degradation	98.88%		120		3	[499]
Copper sulfide	Orange carbon	Photocatalytic degradation	99.8%		60			[500]
SnO_2_/Cu_*x*_S/TiO_2_	Phenol	Photocatalytic degradation	100%	9	360			[501]
Cu_*x*_S/TiO_2_ composites	Methyl orange	Photocatalytic degradation	99%		300			[502]
	Methylene blue		99%		180			
CuS-TiO_2_ composites	Methylene blue	Photocatalytic degradation	94%		180		4	[503]
CuO-CuS core–shell nanowires	Methylene blue	Photocatalytic degradation	89%		240			[504]
ZnO/CuS heterostructures	Methylene blue	Photocatalytic degradation	87%	7	30			[505]
CuS-CdS	Methylene blue	Photocatalytic degradation	99.97%		10		5	[506]
Copper sulfide nanocrystals/graphene nanocomposites (CuS/GR)	Methylene blue	Photocatalytic degradation	94%		80			[507]
g-C_3_N_4_/CuS nanocomposites	Rhodamine B	Photocatalytic degradation	99.6%		60		3	[508]
	Methylene blue		99%		120			
CuS-rGO nanocomposite	Congo red	Photocatalytic degradation	98.76%	5	90		5	[509]
Cu_2_O@Cu_7_S_4_ core–shell micro/nanocrystals	Methyl orange	Photocatalytic degradation						[510]
Ultrafine CuS nanocrystalline/Fe-doped TiO_2_ nanotubes hybrids	Malachite green	Photocatalytic degradation	100%	5				[511]
Graphene/CuS/ZnO hybrid nanocomposites	Methyl orange	Photocatalytic degradation	99%		30			[512]
CuS/MoS_2_	Methylene blue	Photocatalytic degradation	100%		60			[513]
CuS/ZnS core/shell nanocrystals (NCs)	Rhodamine B	Photocatalytic degradation	100%		30		5	[514]
CuS(5)CdS(5)/TiO_2_	Acid orange 7	Photocatalytic degradation	100%	10	100			[515]
CuS/g-C_3_N_4_ composites	Methylene blue	Photocatalytic degradation			90		5	[516]
Molybdenum sulphide (MoS_2_) nano-petals	Rhodamine B	Photocatalytic degradation	96%		25		5	[517]
Sodium dodecyl sulfate intercalated molybdenum disulfide (SDS-MoS_2_)	Cr(VI)	Sorption	63.92 mg/g	5	732			[518]
Molybdenum disulfide/reduced graphene oxide (MoS_2_/rGO) composites	Pb(II)	Sorption	384.16 mg/g	5	400	25		[519]
Fe(II)-promoted activation of peroxymonosulfate by molybdenum disulfide	Acetaminophen (ACT)	Photocatalytic degradation	94.5%	3	180			[520]
Biochar modified with molybdenum disulfide MoS_2_@biochar	Pb(II)	Sorption	189 mg/g	5	180	25	7	[521]
PVP/MoS_2_	Cr(VI)	Sorption	142.24 mg/g	5	1440	25		[522]

## Functionalized Zero-Valent Metal Nanomaterials

The oxidation state of the metal in the nanoparticles is a key factor for predicting the efficiency of the material. Apart from the oxides and hydroxides of the metals, lower-oxidation-state metal nanoparticles in their pristine state have also been proved to be efficient scavengers of wastewater contaminants. Such metals are generally found in their zero-valent states and are represented as zero-valent nanometals (ZVNMs). Nanoscale elemental metals have been massively utilized for their unique properties. One such example is zero-valent iron nanoparticles. The zero-valent iron nanoparticles tend to be more powerful reducing agents and have been exploited for the removal of various contaminants to date [523].

### Transition Metal/d-Block Nanomaterials

Among the NMs, transition metal nanoparticles have emerged as a promising choice based on their superlative properties. The availability of literature on the utilization of transition metal nanoparticles confirms their efficiency as catalysts. Shi et al. [524] prepared bentonite-supported zero-valent iron nanoparticles by the liquid-phase reduction method. The prepared material was used for the efficient removal of Cr(VI) with sorption efficiency of 90%. Increased sorption of chromium was observed at pH 2, which was explained based on the fact that nZVI corrosion is enhanced at lower pH, diminishing the precipitation of Cr(III) and Fe(III) hydroxides on the iron surface and thus accelerating the sorption process.

Ali and Khan [525] focused on exploiting multiple zero-valent metals including Ni, Cu, and Ag MNPs, loaded onto the surface of sodium polyacrylate (water ball) for catalytic degradation of contaminants. The idea of preparation was to first sorb the metal particles on polymer support and then convert them into their zero-valent state by a reducing agent. The water ball is considered a superabsorbent; i.e., it has the capacity for absorbing material greater than its weight. The prepared substance was used for the removal of 4-nitrophenol (4-NP), 4-aminophenol (4-AP), methyl orange (MO), CR, and MB dyes. The 4-NP was reduced with the addition of NaBH_4_ along with the addition of the catalyst. A redshift in the peak from 318 to 400 nm of 4-nitrophenol was observed with the addition of NaBH_4_. This was attributed to the conjugation when the OH proton of phenol forms the phenolate anion by the activity of NaBH_4_ and the negative charge needs a more electronegative atom to reside on. This is the reason the negative charge is delocalized on the oxygen atom more than the lone pair of electrons of OH and the wavelength shift towards a longer side. The same is the case for 4-AP, which is also reduced by the addition of both NaBH_4_ and the catalyst. The removal of anionic dyes up to 98% was observed by the addition of the catalysts. The results showed that the catalyst is the necessary counterpart of NaBH_4_ for better removal of the contaminants, leading to environmental remediation.

**Fig. 14:**
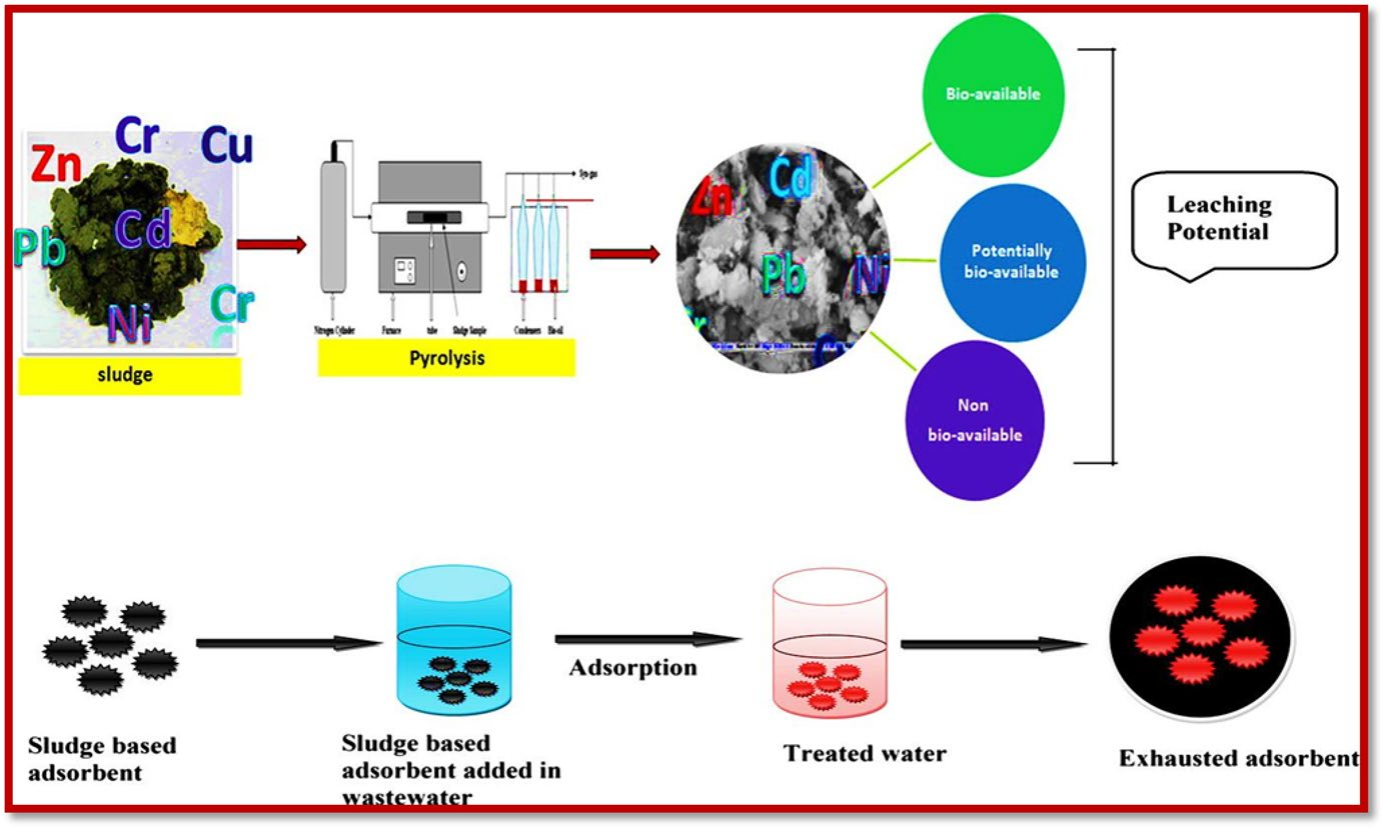
The synthesis and application of sludge-based new adsorbents for the decontamination of wastewater. Reproduced with permission [526], Copyright 2016, Springer Nature

Devi et al. [526] studied the efficiency of plasmonic metal nanoparticles including Ag and Au nanoparticles for the photocatalytic degradation of harmful dyes malachite green (MG) and MB (Fig. 14). The noble metal nanoparticles were prepared using a green approach with extracts of *Hydrocotyle asiatica* as a reducing and stabilizing agent. Firstly, the degradation of MB was performed in the dark using the catalyst followed by solar irradiation in the presence of the catalyst. The results showed that 57% degradation was obtained with no light, and no prominent shift in the wavelength was observed. However, the solar irradiation enhanced the degradation rate to 94%, with a blueshift in wavelength from 617 to 570 nm. No additional peaks were observed, which confirmed that no leuco forms of the dye were formed [527]. In the case of MG, a different behavior was observed due to the different nature of the dye. It is known that MG exists in multiple forms at different pH including chromatic MG^+^ at pH 3–5, protonated MGH^+^ at pH 2, and a colorless carbinol base at pH above 8, all forms having different lambda max. By considering the effect of pH, the reaction was carried out at different pH and the results indicated a shift of wavelength from 617 to 570 nm with the removal of the dye under solar irradiation [528]. The mechanism of the degradation process was explained based on hot electron production on the surface of the Ag nanoparticles by the intraband transition of electrons [529]. The green synthesis of Ag nanoparticles leads to the production of negatively charged surface nanoparticles with zeta potential −34.9 mV. Thus, the cationic dyes can sorb onto its surface through electrostatic interaction, while the electrons may degrade the dyes into simpler products. The blueshift in the lambda max is rendered to the *N*-demethylated intermediates formed during the degradation of dyes. The auxochromic groups are removed, decreasing the intensity of the peaks. It was concluded that the whole mechanism of degradation of dyes follows the demethylation pathway [530].

Shojaei et al. [531] exploited the sorption capacity of zero-valent iron nanoparticles for the removal of Direct Red 81. The results were analyzed by applying the statistical analysis and the effect of various factors on the removal of the dye was considered. It was found that the highest removal was achieved at weakly acidic pH, because, in the weakly acidic range, the zero-valent iron performs well. Lin et al. [532] prepared Fe nanoparticles by a green in situ synthesis method. The prepared iron nanoparticles were utilized for the concurrent removal of Pb^2+^ and rifampicin and the results showed that removal efficiency of the prepared nanoparticles was obtained to be 100% and 91.6% for Pb^2+^ and rifampicin, respectively. The Fe nanoparticles were prepared to utilize the *Euphorbia cochinchinensis* leaf extract. The polyphenols and caffeine present in the leaf extracts assisted as a reducing agent as well as a capping agent for the prepared Fe nanoparticles thus inhibiting the agglomeration probability. Two possible strategies were build-up for explaining the simultaneous removal of Pb^2+^ and rifampicin. Pb^2+^ ions tend to sorb onto the surface of the Fe nanoparticles based solely on the sorption activity of the Fe nanoparticles, while the rifampicin interacted positively with the functional groups present on the surface of the nanoparticles.

### Normal/p-Block, s-Block Nanomaterials

In addition to the great variety of transition metal-based ZVNMs, those of s- and p-block elements in particular have also been utilized for their applicability as ZVNMs for the removal of contaminants from wastewater bodies. The greater utilization of ZVNMs is because they offer more advantages than the limitations of their use [533]. Lin and Lin [534] designed zero-valent aluminum (ZVAl) nanoparticles via washing of the Al with acids. The finally obtained ZVAl was used for the removal of bromate ions from the water. The removal was partially based on the reduction of bromate to bromide and partial sorption of the bromated ions on the surface of the ZVAl. The results showed complete removal of bromated from 78.1 μmol/L solutions at the optimized operating conditions.

Fu et al. [535] prepared a bimetallic material based on aluminum and iron as (Fe/Al) bimetallic particles for the removal of Cr(VI). The morphological studies showed that in the bimetallic particles, iron was deposited onto the surface of the aluminum. The removal efficiency for Cr(VI) was noted as 93.5%, and it was attributed to the high surface area of the bimetallic nanoparticles. Pouretedal et al. [536] prepared zero-valent tin nanoparticles using a reducing agent and utilized them for the photocatalytic degradation of MB dye. The obtained zero-valent tin (ZVT) nanoparticles presented the highest degradation of > 95% of MB at the optimized conditions of operation. The zero-valent Sn is a strong reducing agent in basic solutions with *E*_o_ = −0.91 and −0.93 V. Furthermore, the most important factor influencing *k*_app_ is the surface area of the zero-valent metal, which acts as a strong reducing agent in the basic solutions, thereby reducing the MB dye (Table 7).

**Table 7:** Zero-valent metal-based nanomaterial in wastewater remediation

Nanomaterial	Contaminant	Mechanism	Sorption/catalytic capacity	Conditions studied			Cycles	References
				pH	Time	Temp °C		
Zero-valent iron/Fe_3_O_4_/Fe(II) system (hZVI)	Molybdate	Sorption	97%		2880			[537]
Sulfidated nanoscale zero-valent iron (S-nZVI)	Molybdate	Sorption	98.2%	7	60	25		[538]
Zero-valent iron combined with petroleum coke (CZVI)	Organic compound	Sorption	90.9%	8.5	3360			[539]
Zero-valent iron (Fe^0^) supported by activated carbon (NZVI/AC)	Molybdenum (Mo)	Sorption	90%	4.5	540	25		[540]
Nanoscale zero-valent iron (NZVI) particles	Metronidazole (MNZ)	Sorption	99%	5	5			[541]
Iron-nickel	Tributyl phosphate	Degradation	100%		100			[542]
Zero-valent iron nanoparticles (nZVI)	Microcontaminants	Degradation						[543]
Nanoscale zero-valent iron (nZVI) supported on layered double hydroxide (LDH) composite (LDH@nZVI)	U(VI)	Sorption	176 mg/g	5	300	25	6	[544]
Zero-valent iron (ZVI)	Heavy metals	Sorption	Up to 99%					[545]
Nanoscale zero-valent iron nanoparticles (nZVI)	Plutonium		77%	6	240			[546]
	Uranium		99%					
Zero-valent iron nanoparticle (nZVI)	Citric acid	Degradation	Upto 90%		120			[547]
	Tartaric acid							
	Oxalic acid							
Nanoscale zero-valent iron composite (AT-nZVI)	Cr(VI)	Sorption	90.6%		840			[548]
Nanosized zero-valent iron (nZVI)	Co^2+^	Sorption	172 mg/g		1440		8	[549]
Nanoscale zero-valent iron (SBC-nZVI)	Cr(VI)	Sorption	100%	3	180			[550]
ZVI-based Fenton oxidation processes (ZVI-ZVI/H_2_O_2_)	2,4-dinitroanisole (DNAN)	Degradation	81.3 ± 3.6%	7.2	360			[551]
	2,4-dinitrophenol (DNP)		80.6 ± 1.8%					
	2,4-dinitrochlorobenzene (DNCB)		90.9 ± 3.5%					
Nanoscale zero-valent copper (nZVC)	Azo dyes	Photocatalytic degradation	Up to 90%		240			[552]
Zero-valent copper (ZVC)	Diethyl phthalate (DEP)	Degradation	120	2.5				[553]
Zero-valent copper-catalyzed peroxymonosulfate system	Naproxen	Degradation	Up to 100%	3–7	10	25		[554]
	Bisphenol S							
	Ibuprofen							
Ozone assisted with Cu(0)	Aniline	Degradation	98%		24			[555]
Zero-valent copper nanoparticles (nZVC)	Reactive blue 4	Photocatalytic degradation	90%		15	25		[556]
Zero-valent copper (ZVC)/O_2_/STPP system	p-nitrophenol (PNP)	Photocatalytic degradation	100%	8.1	120			[557]
Zero-valent aluminum (ZVAl)–acid system	Bisphenol A (BPA)	Photocatalytic degradation	75%	1.5	720			[558]
Zero-valent aluminum	Cr(VI)	Photocatalytic degradation	80%			45	5	[559]

## Functionalized Metal Hydroxide Nanomaterials

Metal hydroxides can be categorized as strong bases composed of hydroxide ions and the respective metal ions. The metal hydroxides have recently captured the attention of researchers for their exceptionally attractive properties including high conductivity, thermal and mechanical stability, and flammability [560]. Advanced innovation in this respect is the layered double hydroxides, which offer more refined properties including negligible toxicity, high anionic exchange capacity, and pH-dependent solubility, making them a good choice for environmental restoration purposes [561, 562].

Nie et al. [563] designed a photocatalyst by the combination of Ca(OH)_2_ and peroxymonosulfate (PMS). The prepared Ca(OH)_2_/PMS catalyst was used for the photocatalytic degradation of bisphenol A and phosphate ions simultaneously. The proposed pathway given for the removal of BPA and P showed that superoxide radical (O_2_^−^) and singlet oxygen (^1^O_2_) rather than sulfate (SO_4_^**·**−^) or hydroxyl (HO^**·**^) were the predominant ROS responsible for the degradation of contaminants. The results showed 89.5% BPA and 98.9% P degradation.

Lee et al. [564] developed rice husk (RH)-derived biochar functionalized with Mg/Al-calcined layered double hydroxides (RHB/MgAl-CLDHs) via the co-pyrolysis of MgAl-LDH preloaded RH. The designed RHB/MgAl-CLDHs were used as a sorbent for the successful removal of phosphate from an aqueous solution. The results showed phosphate removal efficiency of up to 97.6% by the prepared sorbent. The obtained results were achieved with the pseudo-second-order model and Sips model, respectively, revealing chemisorption. Koilraj et al. [565] attempted to prepare arginine/lysine-functionalized MgAl LDHs through a one-pot synthesis strategy. The prepared material was used as a sorbent for the removal of Co^2+^ ions. The results presented the highest sorption capacity of 1.159 and 1.170 mmol/g for the LDHs functionalized with lysine and arginine, respectively. The Co^2+^ ions sorption was justified based on the fact that the amino functionalization of the layered double hydroxides tends to form a chelation complex, thereby enhancing the ability for take-up of Co^2+^ ions. The fact that Co^2+^ ions form diamine-like coordination increases its sorption capacity compared with other metal ions. Sadeghalvad et al. [566] tailored a sorbent by loading metal double hydroxides onto the surface of waste rock of iron ore mine (metasomatic rocks) for the removal of sulfate ions. The mechanistic studies confirmed the monolayered chemisorption with the combination of the film-mass transfer and internal diffusion. The maximum sorption capacity obtained was 41.43 and 53.07 mg/g for Mg–Al and Ni–Al metasomatic, respectively (Table 8).

**Table 8:** Metal hydroxide-based nanomaterial in wastewater remediation

Nanomaterial	Contaminant	Mechanism	Sorption/catalytic capacity	Conditions studied			Cycles	References
				pH	Time	Temp °C		
Mg/Fe layered double hydroxide loaded with Magnetic(Fe_3_O_4_) carbon spheres (MCs@Mg/Fe-LDHs)	Pb(II)	Sorption	3.66 mmol/g	7	3000	25	3	[567]
	Cu(II)		5.33 mmol/g					
Ferric hydroxide/graphene oxide	Arsenate	Sorption	95%	4				[568]
Aluminum hydroxide–coated nanoscale zero-valent iron (NZVI@Al(OH)_3_)	4-nitrophenol (4-NP)	Sorption	96.3%	8.3	10	24		[569]
Surfactant-coated aluminum hydroxide [surfactant-Al(OH)_3_]	Sodium dodecyl sulfate (SDS)	Degradation	Up to 99%	7		25		[570]
	Sodium bis(2-ethylhexyl)sulfosuccinate (AOT)							
	Sodium oleate							
Aluminum hydroxides	Herbicide 2-(2,4-dichlorophenoxy)propanoic acid (2,4-DP)	Sorption	93%	7	240	30		[571]
Chitosan/Al(OH)_3_·(CS/Al(OH)_3_)	Fluoride	Sorption	Up to 80%	4	90			[572]
Aluminum hydroxide	Fluoride	Sorption	95%					[573]
Calcite sludge-aluminum hydroxide(CAl)	Bisphenol A	Sorption	83.53 mg/g	3			5	[574]
	Ibuprofen		34.96 mg/g					
Aluminum hydroxide gel-coated nanoscale zero-valent iron (AHG@NZVI)	Tetracycline (TC)	Sorption	98.1%	6.5	70	25		[575]
Calcium hydroxide	Polychlorinated biphenyls (PCBs)	Sorption	94%			600		[576]
Calcium hydroxide	Hydrogen chloride							[577]
Calcium hydroxide nanorods (CHN)	Fluoride	Sorption	450 ± 10 mg/g	6.5	45			[578]
Calcium hydroxide-coated dairy manure-derived biochar (Ca-BC)	Phosphate	Sorption	95%	8.5	4320			[579]
Calcium hydroxide	Lignin	Sorption	70%	5	10	25		[580]
Granular activated carbon-supported magnesium hydroxide (Mg-GAC)	Cd(II)	Sorption	3.47 mg/g	6	480	25		[581]
Modified bentonite with magnesium hydroxide Mg(OH)_2_	Phosphate	Sorption	> 54%	7	45	120		[582]
Mg(OH)_2_	Fluoride	Sorption		5				[583]
Mg(OH)_2_ & Ba(OH)_2_	Metals	Sorption						[584]
NZVI surface coated with Mg(OH)_2_ shell (NZVI@Mg(OH)_2_)	Cr(VI)	Sorption	97.8%	7.5				[585]
Mg(OH)_2_	Phosphorus	Sorption	588.4 mg/g					[586]
Magnesium hydroxide	Oil	Sorption	10 959 mg/g			25	5	[587]
Lignin-Mg(OH)_2_ nanocomposite	Ni^2+^	Sorption	Up to 100%		1500			[588]
	Cd^2+^							
	Pb^2+^							
Cellulose acetate/Mg–Al layered double hydroxide (Mg–Al LDH) nanocomposite membranes	Diclofenac sodium (DS)	Sorption	Up to 100%					[589]
	Tetracycline (TC)							

## Functionalized Silsesquioxane-Based (Silica-Based) Nanomaterials as Sorbents for Contaminant Removal

Other NMs worth mentioning are silica-based NMs (SNMs), also termed silsesquioxane-based NMs with respect to the siloxane rings incorporated in the framework of the NMs. SNMs are obtained by applying high temperature to the silica, accumulating the siloxane rings in the multiple-structured NMs [590]. These NMs are used in various fields due to their extraordinary properties including cytotoxicity, high porosity, high mechanical strength, cost-effectiveness, and biocompatibility [591]. The fabrication of SNMs with additional functionalities resulting in one-, two-, and three-dimensional structures governs its mechanical strength, enhancing the activity of the FSNMs.

Araghi and Entezari [592] designed amino-functionalized silica magnetite nanoparticles (A-S-MNPs) by the coating of sono-synthesized magnetite nanoparticles (MNPs) in a basic medium by SiO_2_. The obtained silica MNPs were then further modified with 3-aminpropyltriethoxysilane (APTES). The estimated particle size of the prepared nanocomposite was 25 nm. The prepared material was then used for the sorptive removal of Reactive Black 5 (RB5) and sodium dodecylbenzenesulfonate (SDBS). The results showed sorption efficiency of 83.33 and 62.5 mg/g for RB5 and SDBS, respectively. Mahmudi et al. [593] prepared dendritic fibrous nano-silica-grafted d-penicillamine. The prepared dPA-DFNS-NH_2_ had a surface area of 78.2m^2^/g, pore volume of 0.13cm^3^/g, and average pore size of 6.7 nm. The prepared material was used as an efficient sorbent for the removal of heavy metals Co^2+^, Ni^2+^, Ag^+^, and Pb^2+^from water samples, with complete removal efficiency. Wang et al. [594] prepared silica nanotubes through an electrospinning and calcination process followed by their modification by sym-diphenylcarbazide (SD-SNTs). The prepared composite was used as an effective sorbent for the removal of Pb(II). The surface functionalization conspicuously enhanced the sorptive efficiency of the material due to the increased possibility for chelation between the imino groups and lead ions (Table 9).

**Table 9:** Silica-based nanomaterials in wastewater remediation

Nanomaterial	Contaminant	Mechanism	Sorption/catalytic capacity	Conditions studied			Cycles	References
				pH	Time	Temp °C		
Mesoporous silica	Co^2+^	Sorption	89%	7	480			[595]
Silica coated β-cyclodextrin polymeric adsorbent	17β-estradiol	Sorption	90%	6.1–6.8	2880		4	[596]
Magnetic silica microrods (R-Fe_3_O_4_@SiO_2_)	Cu(II)	Sorption	322.58 mg/g		30			[597]
	Pb(II)		346.02 mg/g					
	Cr(III)		384.62 mg/g					
	Zn(II)		308.64 mg/g					
	Co(II)		316.46 mg/g					
Ordered mesoporous silica (OMS) incorporated polyvinylidene fluoride (PVDF)	Methylene blue	Sorption	14.5 mg/g				5	[598]
	Cu(II)		1.5 mg/g					
Corn cob silica-alginate beads	Phenol	Sorption	93%		5760			[599]
CuO/ZrO_2_–MCM-41 (CuO@ZM-41)	Cr^6+^	Photocatalytic degradation	100%		30			[600]
Iron-incorporated mesoporous silica	Tetracycline	Sorption	155.76 mg/g	5–7	800	25		[601]
Amino-functionalized SBA-15-NH_2_ ordered mesoporous silica (OMS) materials	Ampicillin	Sorption	333 mg/g	7.4	1500	25		[602]
Silica (SiO_2_)-decorated carbon nanotubes (CNTs) sponge	Oil	Sorption	100%				10	[603]
Mesoporous silica (AMS)	Cd(II)	Sorption	11.54 mg/g	5			5	[604]
	Pb(II)		8.59 mg/g					
Diamine-functionalized mesoporous silica on multi-walled carbon nanotubes (NN-mSiO_2_@MWCNTs)	Cu(II)	Sorption	66.57 mg/g	6.2		25		[605]
All-silica zeolite beta	Perfluorinated compounds	Sorption						[606]
3D organized mesoporous silica (OMS)	As(V)	Sorption	55 mg/g	5				[607]
Silica gel	Polychlorinated diphenyl sulfides (PCDPSs)	Degradation	75.6%	11				[608]
Silica gel modified with p-toluidine formaldehyde resin	Cr(VI)	Sorption	43.47 mg/g	1	300	50		[609]
Silica mesoporous materials of MCM-48 type	Safranin dye	Sorption	62.5 mg/g					[610]

## Quantum Dots, a New-Fashioned Approach for Utilizing Nanomaterials in Wastewater Contamination

Quantum dots (QDs) can be described as semiconductor nanoparticles having size- and composition-dependent electronic and optical properties (optoelectronic properties). The quantum dots are manmade nanoscale crystals that have the tendency to transport electrons [611]. The QDs, nanoparticles of semiconductor materials, are ultra-small, with a size range of 1.5–10 nm. When the size of semiconductors is this small, quantum effects are initiated, limiting the energies at which the electrons and holes (in the absence of e^−^) can exist in particles. As there is a relationship between energy and wavelength, the optical properties of the particle can be tuned depending on its size [612, 613]. Thus the particles can emit or absorb certain wavelengths of light by controlling their size. QDs have been found to possess unique properties including high extinction coefficient and brightness, photo-stability, size-dependent optical properties, and large Stokes shift. Based on the unique chemistry and properties of QDs, they have been used extensively in the fields of electronics, catalysis, medicine, imaging, sensing, and information storage, among others [614]. In this section of the paper, we will discuss QDs of different materials and their applicability with respect to environmental remediation.

### Graphene QDs

Graphene QDs can be described as small fragments of graphene in which electronic transport is observed in all three spatial dimensions. Graphene semiconductor material has a zero bandgap and possesses an infinite exciton Bohr diameter. The confinement can be seen in any of the fragments, but the GQDs possess dimensions in the size range below 20 nm [615]. The GQDs are usually prepared by the cutting or fragmentation of the graphene sheets through the top-down approach. The most attractive properties of the GQDs include their abundant presence, low toxicity, solubility in a variety of solvents, and capacity for further functionalization. These properties enhance the applicability of the GQDs in various fields [616]. Kaur et al. [617] prepared nitrogen-doped graphene quantum dots through a cost-effective thermal pyrolysis process [618]. The N-GQDs exhibited excellent fluorescence with a maximum fluorescence at 440 nm. The prepared chemosensor was analyzed for its selectivity towards the analyte of interest (TNT) by treating it with a mixture of nitro-substituted phenols, metal ions, and other aromatics. The results showed that the fluorescence spectrum of the N-GQDs was greatly quenched as trinitrotoluene was added to the system, while the rest of the aromatics had little or no effect at all on the fluorescence spectrum. These results confirmed the specificity and the sensitivity of the prepared QDs for the detection of TNT. The mechanistic pathway for the quenching between N-GQDs and TNT was explained based on the fact that a spectral overlap was developed between the emission spectrum of N-GQDs and the absorption spectrum of TNT, according to the fluorescence resonance energy transfer (FRET) mechanism, leading to quenching. Another possibility is that an electrostatic interactive force was developed between the OH end of TNT, NH_2_-group, and pyridinic nitrogen of the N-GQDs, eventually resulting in quenching [619]. The excessive presence of TNT led to a redshift in the emission peak due to the high acidity of TNT relative to other contaminants, leading to the formation of a non-fluorescent complex, thus resulting in quenching [620]. These prepared N-GQDs were used as a probe sensor for the detection of an explosive, trinitrotoluene (TNT), and showed excellent efficiency.

Qu et al. [621] also prepared a composite of TiO_2_ nanotubes decorated with graphene quantum dots (GQDs/TiO_2_ NT composites) that exhibits greater photoluminescence quantum yield, leading to excellent optical properties and thus making it a useful photocatalyst. The prepared photocatalyst was used for exploiting its photocatalytic activity for the degradation of MO dye under a UV–Vis irradiation source. The greatest efficiency of 94.64% in 20 min was obtained, which was attributed to the broadened visible light absorption and enhanced photocatalytic activity of the prepared composite. The mechanism for the degradation of MO was explained by the fact that incorporation of GQDs with TiO_2_ led to enhanced absorption ability of TiO_2_ in the visible range, due to the π-state combination of the GQDs and the CB of TiO_2_. Upon photogeneration of electrons from the TiO_2_, the GQDs come forward to capture these electrons, ensuring the separation of e^−^/h^+^ pairs [622]. The up-conversion photoluminescence properties of GQDs cause the conversion of longer irradiation wavelength into a shorter one, and the presence of oxygen on the surface of GQDs captures electrons and oxygen radicals, which then eventually cause the oxidation of MO dye. In addition, holes on the surface of TiO_2_ cause the production of hydroxyl radicals, which completes the degradation of MO into simpler compounds [623].

### Carbon QDs

Another huge class of QDs is carbon quantum dots (CQDs) or fluorescent carbon nanoparticles. CQDs generally exist in quasi-spherical nanoparticle form. They consist of an amorphous to nanocrystalline core of graphitic or turbostratic carbon (sp^2^ carbon) [624]. Another possibility is the presence of graphene and graphene oxide sheets fused together by diamond-like sp^3^-hybridized carbon insertions. The oxidized form of CQDs contains many carboxyl moieties on its surface. The carboxyl moieties may impart a solubility factor to the CQDs and also provide suitable chemically reactive groups which help in further functionalization and surface passivation. Surface passivation using groups such as inorganic, organic, or polymer materials will further enhance the fluorescence properties and solubility of the CQDs [625]. Saud et al. [626] prepared carbon quantum dot/titanium dioxide nanocomposite (CQD/TiO_2_) nanofibers by a hydrothermal method [627]. The prepared nanocomposite was utilized to evaluate its efficiency for the photocatalytic degradation of MB under visible light irradiation and antibacterial activity against *Escherichia coli.* TiO_2_ is known to have excellent photocatalytic properties under UV-irradiation, and the incorporation of CQDs will enhance its photocatalytic efficiency by decreasing the wavelength, promoting the production of electrons in a wider range of visible irradiation. The CQDs act as a sink for capturing the produced electrons and their mobilization. The CQD/TiO_2_exhibited complete degradation of the MB dye in 20 min with an additional property of the reusability of the photocatalyst for up to three cycles. The efficiency was slightly reduced for the third run due to the covering of the active sites of the catalyst, hence minimizing their availability for the photocatalytic operation.

**Fig. 15:**
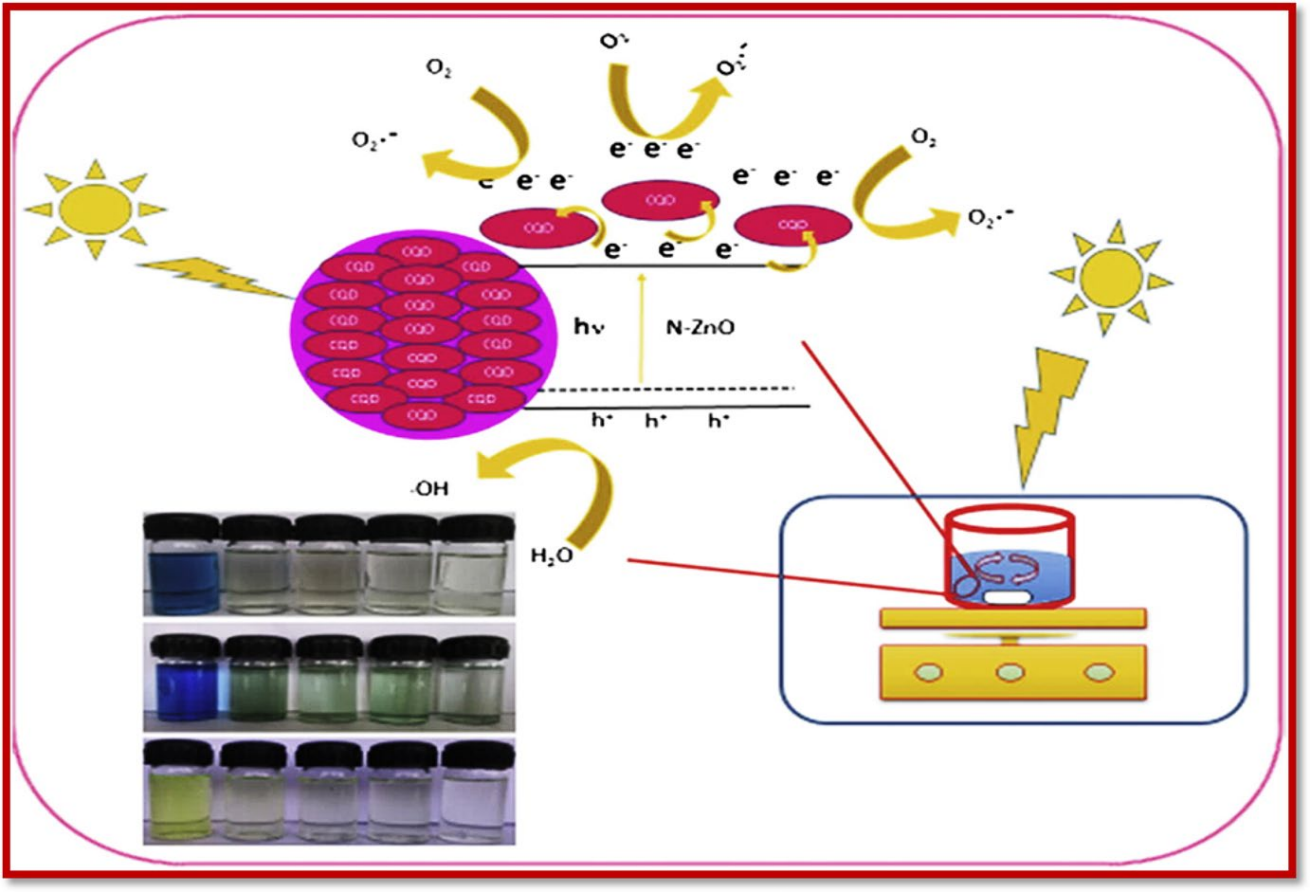
Schematic explanation, experimental setup, and mechanism of CQD/N-ZnO on dyes under natural daylight irradiation. Reproduced with permission [628], Copyright 2015, Elsevier

Muthulingam et al. [628] prepared carbon quantum dots/nitrogen-doped ZnO composites by a simple one-step method, which were utilized for the photocatalytic degradation of commercial dyes including malachite green, MB, and fluorescein. The obtained results showed that the CQD/N-ZnO photocatalyst exhibited 100% removal efficiency for the malachite green in 30 min irradiation under visible light (Fig. 15). For MB, it took 45 min to completely degrade the dye, while in the case of fluorescein, 95% was obtained in 15 min of daylight exposure. The excellent degradation efficiency exhibited by CQD/N-ZnO was attributed to the combined features of the CQDs and ZnO, and inhibition of the recombination of photogenerated electron/hole pairs due to the CQD and nitrogen doping of the ZnO [629]. Once the energy equal to or higher than the bandgap of the photocatalyst is achieved, the photogeneration of electron/hole pairs indicates the beginning of the process. The π-electronic interaction of the carbon with the CB of the ZnO photocatalyst leads to the up-converting property of the QDs, leading to the high absorption of light in a wider wavelength range. A larger number of holes created near the VB of the ZnO may cause the production of radicals, eventually leading to maximal degradation efficiency. The electrons trapped in the CQDs initiate the production of superoxide radicals, while the combination of holes with water molecules tends to produce hydroxyl radicals. These powerful radicals lead to a combinational approach towards the degradation of dye into simpler and less toxic compounds [630].

### Metal Oxide QDs

Many metal oxide-based quantum dots have been prepared to date to take advantage of their useful properties including optical activity, stability, and conductivity. One significant example is titanium oxide QDs. TiO_2_ has been widely studied since the discovery of its photocatalytic water-splitting ability, owing to its desirable properties such as nonhazardous nature, stability, ease of availability, wider bandgap(*E*_bg_ = 3.2 eV for anatase phase), and cost-effectiveness [631]. Gnanasekaranet al. [632] prepared TiO_2_ quantum dots by the sol–gel method [633], and the efficiency of the prepared material was checked by photocatalytic degradation of MO and MB. The bandgap obtained for the prepared TiO_2_ QDs was 3.79 eV in the UV region. Degradation efficiency of 97% was obtained for both MO and MB in 80 min. The UV light irradiation to the mixture of dye and photocatalyst produces electrons in the CB and in the VB of the photocatalyst. The electrons react with oxygen, producing(oxidation) radicals, while the holes react with water(reduction) producing radicals [634]. The combined radicals cause the degradation of dyes into simpler nontoxic compounds completing the process. Kaur et al. [635] prepared a composite of Ag_2_O/TiO_2_ quantum dots (QDs)thus enhancing the photocatalytic efficiency of the bare TiO_2_ QDs. The prepared QDs had a spherical shape with a size of 2–9 nm. The prepared photocatalyst was utilized for the degradation of the fluoroquinolone levofloxacin drug. The excellent photocatalytic efficiency is attributed to the separation of the produced electron/hole pairs due to the enhanced light absorption property due to the incorporation of Ag_2_O with TiO_2_ QDs. The results showed 81% removal of levofloxacin drug utilizing the QDs in 90 min. The removal efficiency was attained based on the sorption as well as the photocatalytic activity of the QDs. The main photocatalytic species involved are the holes, electrons, ^1^O_2,_ and ^**·**^OH. Soodet al. [636] also prepared TiO_2_ quantum dots using the sol–gel method [633] and performed photocatalytic degradation of indigo carmine dye. The unique characteristics properties of TiO_2_ led to the maximum degradation of the said dye making it an attractive process for the removal of the contaminants. The results indicated that 95% of dye degradation was obtained in 60 min of the photocatalytic process in acidic pH at 25 °C (Table 10).

**Table 10:** Quantum dot-based nanomaterial in wastewater remediation

Nanomaterial	Contaminant	Mechanism	Sorption/catalytic capacity	Conditions studied			Cycles	References
				pH	Time	Temp °C		
Carbon quantum dots and reduced graphene oxide layers-modified S@g-C_3_N_4_/B@g-C_3_N_4_ (CRSB) photocatalyst	Chloramphenicol	Photocatalytic degradation	92.4%	6	120	30	5	[639]
Graphene quantum dots-ZnO nanocomposites	Methylene blue	Photocatalytic degradation			180			[640]
	Methyl orange							
Nitrogen-doped carbon quantum dots modified with g-C_3_N_4_ NCDs/DCN	Ofloxacin	Photocatalytic degradation	75.2%	3.5–7.5	120			[641]
	Bisphenol A		47.4%					
	Ciprofloxacin		75.8%					
	Cr(VI)		92.6%					
Sulfur-doped carbon quantum dots (S-CQDs)/hollow tubular g-C_3_N_4_ photocatalyst (HTCN-C)	Tetracycline	Photocatalytic degradation	82.67%		40		5	[642]
BiOBr/CDs/g-C_3_N_4_ composites	Tetracycline	Photocatalytic degradation	82.7%		60		10	[643]
	Ciprofloxacin		81.3%					
Nitrogen-doped CDs/g-C_3_N_4 (_NCDs)	Indomethacin	Photocatalytic degradation	91%		90		6	[644]
Carbon quantum dots with polydopamine (PDA/CQDs)	Methylene blue	Photocatalytic degradation						[645]
	Orange II							
Graphitic carbon nitride nanorods decorated with graphene quantum dots (GQDs/g-CNNR)	Oxytetracycline	Photocatalytic degradation	80%		120		5	[646]
ZnO sensitized by carbon quantum dots (L-CQDs/ZnO)	Phenol	Photocatalytic degradation	100%		330		10	[647]
SnO_2_ quantum dots decorated on 2-D material g-C_3_N_4_	Rhodamine B	Photocatalytic degradation	95%		60			[648]
SnS_2_ modified with nitrogen-doped carbon quantum dots (N-CQDs/SnS_2_ Composite)	Cr(VI)	Photocatalytic degradation	100%		25		6	[649]
MnO_x_ quantum dots dispersed on N-doped porous carbon shells (denoted as MnO_x_/N-HPCS)	Bisphenol A	Photocatalytic degradation	99%		20	25	4	[650]
Phenylhydrazine-modified carbon quantum dots	Methylene blue	Photocatalytic degradation	94.3%		60			[651]
CDs-N-TiO_2-x_ nanocomposite	Cr(VI)	Photocatalytic degradation	94%	5.36	60			[652]
PVA/CQDs	Methylene blue	Sorption	97%	12	40		5	[653]
Nitrogen-doped carbon quantum dots with g-C_3_N_4 (_NCQD/g-C_3_N_4)_	Methylene blue	Photocatalytic degradation	91.2%		180		3	[654]
Nitrogen-doped graphene quantum dots	Methylene blue	Photocatalytic degradation	93%		60			[655]
α-Bi_2_O_3_/C-dots	Indigo carmine	Photocatalytic degradation	86%	6	120		3	[656]
	Levofloxacin		79%					
Nitrogen-sulfur-doped carbon quantum dots N,S-CQDs/TiO_2_ nanocomposite	Diclofenac	Photocatalytic degradation	62.3%		120			[657]
Carbon quantum dots modified with graphitic carbon nitride	Carbamazepine	Photocatalytic degradation	100%	7	60	25	4	[658]
Biomass-derived carbon quantum dots	Methylene blue	Photocatalytic degradation	99.5%		130			[659]
SnO_2_ quantum dot encapsulated carbon nanoflake (SnO_2_–CNF)	Bisphenol A	Photocatalytic degradation	98%	6	60		3	[660]
Ag-doped SnO_2_ quantum dots	Rhodamine B	Photocatalytic degradation	97.5%		120		7	[661]
Bismuth (Bi)-doped tin oxide (SnO_2_) quantum dots	Rhodamine B	Photocatalytic degradation	98.2%		100		5	[662]
	Ciprofloxacin hydrochloride		92.13%		90			
NiFe_2_O_4_/SQD	Rhodamine B	Photocatalytic degradation	98%		105			[663]
Carbon quantum dots implanted CdSnanosheets (CQD/CdS-NSs)	Cr(VI)	Photocatalytic degradation	94%		10		3	[664]
SnO_2_ quantum dot/gold (SQD/Au) nanocomposites	Methylene blue	Photocatalytic degradation	99%		150		3	[665]
	Rhodamine B		99%		200			
	Methyl orange		93.5%		180			
C_3_N_4_/AgI/ZnO/CQDs (PGCN)	2,4-Dinitrophenol	Photocatalytic degradation	98%		120		10	[666]
Ternary carbon quantum dots (CDs)/Bi_2_MoO_6_ (BMO)/graphitic carbon nanofibers (GNFs) composites (CDs/BMO/GNFs)	Rhodamine B	Photocatalytic degradation	99.4%		70			[667]
CeO_2_ QDs/BiOX (X = Cl, Br) heterojunctions	Tetracycline	Photocatalytic degradation	97%		120		6	[668]
	Cr(VI)							
Ag-SnO_2_ quantum dots(QDs)/silver phosphate (AgSn/AgP) composites	Carbamazepine	Photocatalytic degradation	63.6%		120		3	[669]
Mn-doped ZnS quantum dots capped by L-cysteine (Mn@ZnS/L-cyst)	4′,5′-Dibromofluorescein dye	Photocatalytic degradation	97%	5.5	30			[670]
Carbon quantum dots modified with chitosan (CH-CQDs)	Cd^2+^	Sorption	112.4 mg/g	8	30	25		[671]
Amine-functionalized graphene quantum dots	Methyl orange	Photocatalytic degradation	99%		120		4	[672]
Graphene quantum dots with silver NPs (GQDs/Ag NPs)	Rhodamine B	Photocatalytic degradation			540			[673]
Black TiO_2−x_/N-doped graphene quantum dots (BTNG)	Rhodamine B	Photocatalytic degradation	100%		30			[674]
Nitrogen-doped graphene quantum dots (NGQDs)-BiVO_4_/g-C_3_N_4_ *Z*-scheme heterojunction	Tetracycline	Photocatalytic degradation	91.5%		30		4	[675]
Carbon quantum dots/CdS quantum dots/g-C3N4 (CDs/CdS/GCN) photocatalysts	4-Nitrophenol	Photocatalytic degradation	95%		120		4	[676]
p-type phosphorus-doped graphene quantum dots (P-GQDs)	Methyl orange	Photocatalytic degradation	95.5%		14			[677]
N-doped graphene quantum dots (NGQDs)	Methylene blue	Photocatalytic degradation	98%		150			[678]
Bi(III) containing oxides with quantum dots (QDs)	Cl^−^ removal in leachate		66.1%	1	480			[679]
Carbon quantum dots modified potassium titanate nanotubes (CQDs/K_2_Ti_6_O_13_) composite	Amoxicillin	Photocatalytic degradation	100%	6	90	25		[680]
Nitrogen–phosphorus-doped fluorescent carbon dots (NP-CD)	Cr(VI)	Photocatalytic degradation	100%		110			[681]
N-doped carbon quantum dots/TiO_2_ (NCQDs/TiO_2_)	Methylene blue	Photocatalytic degradation	86.9%		420			[682]
ZnS quantum dots	Methyl violet	Photocatalytic degradation	95%	12	120			[683]
Zinc oxide quantum dots/CuO NSs	Tetanus toxin	Photocatalytic degradation	Up to 90%			25		[684]
(CdS–CdSe)/TiO_2_-NTAs	Methyl orange	Photocatalytic degradation	95.1		120		3	[685]
ZnS quantum dots impregnated-mesoporous TiO_2_ nanospheres	Methylene blue	Photocatalytic degradation	100%		32			[686]
CdTeSe Quantum Dots (QDs)	Rhodamine B		61%	12	1440			[687]
Graphene quantum dots (GQDs) infilled titanium dioxide (TiO_2_) nanotube arrays (NTAs) hybrid	Methylene blue	Photocatalytic degradation	99.8%		180		10	[688]
Cadmium selenide/graphene quantum dots (CdSe/GQDs)	Methylene blue	Sonocatalytic degradation	99%	9	90		5	[689]
Graphene quantum dots	Oxamyl	Sorption	125 mg/g	8	25	20		[690]
N-doped reduced graphene quantum dots	Rhodamine B	Sorption	24.62 mg/g	7	720			[691]
Fe_3_O_4_/hydroxyapatite/graphene quantum dots (Fe_3_O_4_/HAP/GQDs)	Methyl orange	ICP-AES	37.99 mg/g				7	[692]
	Methylene blue		15.35 mg/g					
	Cu		83–104%					
Layered double hydroxide–carbon dot composite	Methyl blue	Sorption	185 mg/g		60	25		[693]
CQDs@PAFPnanobiosorbent	U(VI)	Sorption	95–98%	5			4	[694]
Graphene quantum dots (GQDs) immobilized onto the NiFe_2_O_4_-halloysite nanotubes (NiFe_2_O_4_-HNTs)	Pb(II)	Sorption	42.02 mg/g			25		[695]
PVA/CMC-B@GO/Fe_3_O_4_/GQD (L)	Methylene blue	Sorption	1000 mg	8	240		4	[696]

### Metal Sulfide QDs

Metal sulfides constitute a distinct class of quantum dots. Upgraded metal sulfide QDs have proved to be more effective in their respective activity based on their advanced properties than their simple sums. Rajabi et al. [637] performed a comparative study to evaluate the photocatalytic efficiency of functionalized ZnS QDs and iron oxide (Fe_3_O_4_) MNPs for the removal of Victoria blue R dye. Both photocatalysts were prepared by a simple chemical precipitation method, while the surface modification was performed using 2-mercaptoethanol and sodium dodecyl sulfate. The particle size calculated for the ZnS QDs and the MNPs was 1–3 nm and 50–80 nm, respectively. The results obtained showed 95% and 65% removal efficiency for MNPs and QDs, respectively. Zinc sulfide quantum dots doped with Fe(III) were prepared for the removal of methyl violet [638]. The bandgap calculated for the prepared photocatalyst was > 4.58. About 98.8% decolorization was obtained using the prepared photocatalyst, showing the extraordinary activity of the doped photocatalyst.

## Miscellaneous Functionalized Nanomaterials

Apart from the FNMs discussed above, several other NMs have demonstrated attractive properties, such as dendrimers [697], nanoclays [698], ceramics [699], and mesoporous materials [700]. Sohail et al. [701] studied the preparation of polyamidoamine (PAMAM) dendrimers for the removal of nickel ions. Dendrimers have special qualities including radial symmetry and homogeneity, having a monodisperse and well-defined structure, with tree-like branches characterized by terminal poly-functionality. The dendrimers were synthesized using the divergent method, by initiating at the core, leading towards the periphery following two basic operations. The first one is the coupling of the monomer, while in the second step the de-protection/transformation of the monomer end group occurs. It creates a new reactive surface functionality that couples a new monomer in a similar way as that of the solid-phase synthesis of peptides or oligonucleotides. A particle size of 827 nm was found for the prepared zero-generation dendrimer, and the sorption capacity for the prepared material was 98.6 mg/g.

Hayati et al. [702] studied the efficiency of a poly(propylene imine) (PPI) dendrimer against the decantation of Direct Red 80 (DR80) and Acid Green 25 (AG25) dyes. Maximum removal efficiency was obtained for the removal of both dyes. Analysis of the data showed that the sorption results best fitted the Langmuir isotherm, which predicts the monolayer sorption of the dyes onto the dendrimer surface.

Almasri et al. [703] studied the preparation of hydroxyl iron-modified montmorillonite (HyFe-MMT) nanoclay through the wet chemical synthesis method. The prepared HyFe-MMT nanoclay was used for the removal of arsenite(III). The BET-analyzed surface area of MMT nanoclay was found to be in the range of 277–355 m^2^/g. The mechanistic approach confirmed that the sorption of arsenite took place through both the outer-sphere (physisorption) and inner-sphere complexes (chemisorption) at the hydroxyl iron nanoclay surface. The sorption capacity obtained for the arsenite removal was 3.85 mg/g. Nikkhah et al. [704] explored the synthesis of a Cloisite 20A nanoclay-modified polyurethane foam structure and used it for the removal of oil from an oil–water system. Sorption capacity of 21.5 mg/g was achieved by the prepared sorbent. Narwade et al. [705] explored the synthesis of hydroxyapatite (HAp) by the wet-chemical precipitation method and used it as a sorbent for phenol removal. Sorption capacity of ~ 64 mg/g was achieved, confirming the efficiency of the prepared substance. Phenol tends to form phenoxide ions when dissolved in water. At acidic pH, a variety of reactions take place between the phenoxide ions and the surface of the CNF–HAp films.

## Mechanism of Pollutant Remediation

Generally, the mechanism of the interaction of heavy metal ions with the sorbents can be discussed in terms of the chelation of the metals with anionic functional groups present on the surface of the sorbent [706]. Heavy metals can be removed through sorption only if they are completely entrapped by the chelating agents present on the surface of the sorbent. Most of the chelating groups contain carboxylic acids which are directly connected to one or more nitrogen atoms. These groups also hinder the precipitation of the metals, inhibit metal ion catalysis, and ensure the availability of metal ions in the reaction system [707]. The key groups taking part in metal chelation are the carboxyl and amino groups.

The pH also affects the uptake of the metals by the chelating groups. If the pH is low, ligands tend to be associated with hydronium ions and inhibit the metal cation approach. Also, at lower pH, the carboxylic groups are mostly not dissociated, although they are part of the complexation reactions. The chelation mechanism follows the formation of quinquedentate, hexadentate, and sometimes distorted structures [708]. In the case of the dye molecules, they generally tend to follow the electrostatic interaction with that of the adsorbent surface. The electrostatic interaction can be affected by properties such as surface charge, degree of ionization, and speciation. In general, the sorbent surface may have acidic or alkaline characteristics, which may interact with the dye contaminants through the pH effect. At lower pH, the surface of the sorbent is protonated, bearing a positive charge. This positive charge will electrostatically attract the negatively charged dyes, causing their removal from the aqueous solution. In the case of a basic medium, the surface of the sorbent may be completely ionized, causing repulsive forces with the dye anions [709].

The interaction of pharmaceutical compounds with sorbent molecules can be explained based on hydrogen bonding. The hydrogen bonding occurs when the hydrogen bonding donor groups on the surface of the sorbent interact with those of the hydrogen bonding acceptor atoms on the surface of the contaminants. Generally, the functional groups containing nitrogen and oxygen atoms act as the H-acceptors, which may interact with the –OH or phosphorus-containing groups. Other factors involved in the interaction include n–π, and π–π interaction is also common, which is a specific and non-covalent interaction and exists between electron-rich and electron-poor compounds [710].

### Mechanism of Sorption

Sorption is a surface phenomenon in which the sorbate molecules attach to the surface of the sorbent through either physical or chemical interactions. The physical interaction, or physisorption, may be driven by van der Waals forces or electrostatic interactions, which are fast, reversible, and result in multilayer formation on the surface of the sorbent. On the contrary, chemical interaction, or chemisorption, exhibits strong covalent bonding which is slower and requires activation energy, is irreversible, and produces a monolayer on the surface of the sorbent. The sorption phenomenon is greatly affected by the reaction conditions such as pH, temperature, time, and sorbate concentration and dosage. The sorption of contaminants can be analyzed by the amount of contaminant sorbed per unit mass of sorbent (*q*_e_) and the residual left (*C*_e_) at equilibrium conditions. Different isotherm models have been designed to explain the mechanism and bonding interactions [711, 712].

### Sorption Isotherm Models

The sorption results can be further evaluated by applying the sorption isotherm models, which are discussed in detail as follows. The Langmuir isotherm model is based on the conditions where the surface of the sorbent is homogeneous, providing equal binding sites, and the sorbate molecules tend to form a monolayer on the surface. The following equation is designed based on the Langmuir model (Eq. 9):

9 $$\frac{{C}_{e}}{{q}_{m}}=\frac{1}{{K}_{L }{q}_{\mathrm{max}}}+\frac{{C}_{e}}{{q}_{\mathrm{max}}}$$

where *C*_e_ stands for the equilibrium concentration (mg/L), *q*_m_ is the amount sorbed per unit mass of sorbent (mg/g), and *K*_*L*_ is the Langmuir equilibrium constant related to the heat of sorption [713].

Another model to explain the sorption process is the Freundlich isotherm model, which is generally applied to the multilayer sorption of sorbate molecules onto a heterogeneous surface. The linear equation of the Freundlich isotherm model is as follows (Eq. 10):

10 $${\mathrm{log}}_{{q}_{m}}=log{K}_{f}+\frac{1}{n}\mathrm{log}{C}_{e}$$

where *q*_m_ (mg/g) indicates the molecules sorbed (amount) onto the sorbent surface, *Ce* (mg/L) denotes the equilibrium concentration, and *n* and *K*_f_ are the Freundlich constant and Freundlich exponent, respectively. *K*_*f*_ (mg/g) is the sorption capacity, while *n* shows the degree of the surface heterogeneity and conveys the distribution of the sorbed molecules on the sorbent surface. A higher value of *n* indicates a positive rise in sorption and gives the intensity of sorption [714].

Yet another isotherm model, called the Temkin isotherm, shows the effect of the heat of sorption, which is inversely related to the sorption and monolayer formation. The linear decrease in the sorption heat is caused by the interaction between the sorbent and sorbate molecules. The Temkin isotherm model is expressed in the following form (Eq. 11):

11 $${q}_{e}=\left(\frac{RT}{{b}_{T}}\right)ln\left({AC}_{e}\right)$$

Here, *T* is the temperature (K), *R* is the ideal gas constant (8.314 J/mol/K), *b*_*T*_ represents the Temkin constant (J/mol), which depends on the heat of sorption, and *A* is the equilibrium sorption constant, corresponding to the maximum sorption energy (L/mg) [715].

The sorption phenomenon can also be described by the Brunauer–Emmett–Teller isotherm model, which is generally based on the assumptions of the Langmuir sorption isotherms and is widely used to calculate the surface area and porosity of the system. This model is generally applied to gas–solid systems [716]. The equation form of the model can be represented as Eq. (12):

12 $$ln {q}_{e}$$

The Redlich–Peterson isotherm is a combined form of the Langmuir and Freundlich isotherm models, taking hints from the assumptions of both the models. This model is applied to systems having both homogeneous and heterogeneous surfaces of sorbents over a wide range of sorbate concentrations. At higher sorbate concentrations, Freundlich isotherm assumptions are followed, while at a lower concentration, Henry’s law is obeyed [717]. The equation, Eq. (13), for this model is represented as follows:

13 $$\mathrm{ln}{q}_{e}=\left(\frac{1}{{n}_{H}}\mathrm{ln}{K}_{H}\right)-\left(\frac{1}{{n}_{H}}ln\frac{1}{{C}_{e}}\right)$$

where *K*_*RP*_ (L/g) and *α* (L/mg) are Redlich–Peterson constants, and *β* is an exponential value in the range between 0 and 1. If the *β* value is 0, the isotherm behaves as Langmuir's model, while it obeys Henry’s law when the *β* value is 1.

### Mechanism of Photocatalysis

A general definition of photocatalysis can be derived from the reported literature, as the breakdown of organic pollutants in a spontaneous reaction from the principles of thermodynamics. As its name suggests, photocatalysis refers to the lysis/breakdown/degradation of pollutants with the aid of light and a catalyst [718–720]. The mechanism of photocatalysis can be divided into four major steps:

- Light is absorbed, which generates electron/hole pairs.
- The excited charges are separated.
- The electrons and the holes are transported to the surface of the photocatalyst.
- The redox reactions occur on the surface by utilizing the charges.

In the third step, the electrons/holes may recombine and scatter the harvested light energy in the form of heat (non-radiative recombination) or light (radiative recombination). The remaining photogenerated charges present on the surface of the catalysts tend to carry out the redox reactions, depending on the acceptor or donor properties of the absorbed species [721, 722].

## Conclusion

Worldwide concern related to wastewater contamination has driven researchers to develop advanced, rapid, and accurate methods for the removal of a variety of contaminants primarily emerging from industrial sources. These contaminants have hazardous effects on living organisms, requiring the development of efficient means of decontamination. This quest has led researchers to the use of NMs based on their unique properties including very small size and high surface area-to-volume ratios. NMs have revolutionized conventional techniques used for the treatment of contaminants such as sorption and photocatalysis by improving and enhancing the productivity of these techniques. Innovation in the field of nanotechnology has been achieved by the functionalization of the pristine NMs, thus incorporating the useful properties of multiple materials to provide better results. This review covers the available reports on the removal of contaminants by FNMs for the past 10 years, highlighting their bright future.
