# Correction to: Covalent and Non-covalent Functionalized Nanomaterials for Environmental Restoration

**DOI:** 10.1007/s41061-022-00405-6

**Published:** 2022-09-02

**Authors:** Shizhong Zhang, Sumeet Malik, Nisar Ali, Adnan Khan, Muhammad Bilal, Kashif Rasool

**Affiliations:** 1 Key Laboratory for Palygorskite Science and Applied Technology of Jiangsu Province, National and Local Joint Engineering Research Center for Mineral Salt Deep Utilization, Huaiyin Institute of Technology, Huai’an, 223003 China; 2 Institute of Chemical Sciences, University of Peshawar, Khyber Pakhtunkhwa, 25120 Pakistan; 3 School of Life Science and Food Engineering, Huaiyin Institute of Technology, Huai’an, 223003 China; 4 Qatar Environment and Energy Research Institute, Hamad Bin Khalifa University (HBKU), Qatar Foundation, P.O. Box 5824, Doha, Qatar

## Correction to: Topics in Current Chemistry (2022) 380:44 10.1007/s41061-022-00397-3

In this article the co-author’s name was incorrectly written as Mohammad Bilal.

It should be corrected as Muhammad Bilal.

The original article has been corrected.

